# A method to estimate probability of disease and vaccine efficacy from clinical trial immunogenicity data

**DOI:** 10.1038/s41541-021-00377-6

**Published:** 2021-11-04

**Authors:** Julie Dudášová, Regina Laube, Chandni Valiathan, Matthew C. Wiener, Ferdous Gheyas, Pavel Fišer, Justina Ivanauskaite, Frank Liu, Jeffrey R. Sachs

**Affiliations:** 1 Quantitative Pharmacology and Pharmacometrics, MSD, Czech Republic; 2 First Faculty of Medicine, Charles University, Prague, Czech Republic; 3 MRL IT, MSD Czech Republic, Prague, Czech Republic; 4 MRL IT, Merck & Co., Inc., Kenilworth, NJ USA; 5 Quantitative Pharmacology and Pharmacometrics, Merck & Co., Inc., Kenilworth, NJ USA; 6 AH IT, MSD Czech Republic, Prague, Czech Republic; 7 Biostatistics and Research Decision Sciences, Merck & Co., Inc., Kenilworth, NJ USA; 8 Present Address: Quantitative Sciences, Janssen Research & Development, San Diego, CA USA; 9 Present Address: Department of Informatics and Predictive Sciences, Celgene, a BMS Company, Boudry, Switzerland

**Keywords:** Vaccines, Vaccines, Predictive markers, Clinical trial design, Drug development

## Abstract

Vaccine efficacy is often assessed by counting disease cases in a clinical trial. A new quantitative framework proposed here (“PoDBAY,” Probability of Disease Bayesian Analysis), estimates vaccine efficacy (and confidence interval) using immune response biomarker data collected shortly after vaccination. Given a biomarker associated with protection, PoDBAY describes the relationship between biomarker and probability of disease as a sigmoid probability of disease (“PoD”) curve. The PoDBAY framework is illustrated using clinical trial simulations and with data for influenza, zoster, and dengue virus vaccines. The simulations demonstrate that PoDBAY efficacy estimation (which integrates the PoD and biomarker data), can be accurate and more precise than the standard (case-count) estimation, contributing to more sensitive and specific decisions than threshold-based correlate of protection or case-count-based methods. For all three vaccine examples, the PoD fit indicates a substantial association between the biomarkers and protection, and efficacy estimated by PoDBAY from relatively little immunogenicity data is predictive of the standard estimate of efficacy, demonstrating how PoDBAY can provide early assessments of vaccine efficacy. Methods like PoDBAY can help accelerate and economize vaccine development using an immunological predictor of protection. For example, in the current effort against the COVID-19 pandemic it might provide information to help prioritize (rank) candidates both earlier in a trial and earlier in development.

## Introduction

The protective efficacy of a vaccine (vaccine efficacy, “VE”) is defined as the proportional reduction in risk of disease among vaccinated subjects compared to control (placebo vaccinated) subjects and is often assessed in randomized double-blinded controlled clinical trials^1^. Compared to other drugs and biologics, vaccine trials are particularly costly and lengthy^2,3^. This is due in part to the number of subjects in the trials, the need to enroll many healthy subjects (as opposed to those currently suffering from a disease, who may be more motivated to participate), and observation periods of months to years to accrue the number of disease cases necessary to obtain a sufficiently precise estimate of VE. An overview of statistical methods for VE assessment and alternative trial designs can be found in Chen and Ting^4^.

### What is a CoP?

In some trials, immune response post vaccination is evaluated in addition to the primary clinical endpoint of disease. If a biomarker provides an adequate prediction of protection from disease and is measured sufficiently precisely, then it can be identified as a potential immunological correlate of protection (“CoP”). Biomarkers predictive of protection have been discussed widely in the literature. Authors and public health authorities^5–8^ use terms such as “correlate of protection”, “surrogate of protection”, “immune marker of protection”, “correlate of immunity”, and “correlate of risk” with different definitions and sometimes in directly contradictory ways.

In this work, the term “correlate of protection,” or “CoP,” is used in accordance with the terminology of Plotkin and Gilbert^6^, in which a CoP is a biomarker that can be used to reliably predict VE. This was chosen because the biomarker used in the proposed method can be a mechanistic CoP, a non-mechanistic CoP, an absolute CoP, or a relative CoP. Supplementary Note 9 compares various terminologies.

### Why is CoP important?

Establishing and using CoPs in vaccine clinical development can be valuable for (i) understanding the mechanism of protection, (ii) identifying promising vaccine candidates, (iii) evaluating immunogenicity in small-scale clinical trials, and assisting “go/no-go” decisions (from early to late-stage clinical trials), (iv) reducing size or duration of large-scale clinical trials, (v) providing endpoints for use in bridging clinical trials (e.g., to show different formulations are sufficiently similar or supporting the protection of additional populations), and (vi) providing additional support in the evaluation of vaccines by regulatory and public health authorities. CoPs generally lead to faster development of effective vaccines^9^.

### How can CoP be assessed?

Several approaches have been applied to search for a threshold of the biomarker (immunogenicity measure) that discriminates subjects likely to have disease versus those likely not to have disease^10–14^, or to quantify the continuous relationship between an immune response biomarker and protection from disease^15–18^.

### Continuous (“relative”) CoP versus thresholds (“absolute” CoP)?

Use of the threshold model assumes that individuals who achieve the threshold after vaccination are completely protected. Biomarkers for which this model is valid are termed “absolute correlates”^6^. The relationship is more likely to be continuous, and the majority of biomarkers are “relative correlates” in the sense that higher values are quantitatively more protective than lower values with occasional failures even at high levels and occasional protection at lower levels^6^. Further, even if there were a universal, absolute threshold for protection, assay variability would result in the measured relationship being continuous.

#### Continuous CoP — history

Hobson et al.^19^ first analyzed a quantitative relationship between a biomarker and the likelihood of infection. Recently, Dunning^16^ proposed to use the logistic function of log biomarker value to model the protection curve. Furthermore, he provided a formula to calculate VE using the protection curve and biomarker values of individuals in vaccinated and control groups. Coudeville et al.^20^, building on Dunning’s approach, estimated the level of clinical protection using a random-effects model with covariates (allowing the inclusion of multiple datasets in meta-analytical approach) and included uncertainty in the estimation of the protection curve and distributions of biomarker values to obtain not only a point estimate of VE but also its confidence interval (“CI”)^21^. Other authors^22^ have provided an excellent overview of approaches to surrogate markers and proposed optimizing curve shape using machine-learning methods while maintaining desirable statistical properties; this very general approach minimizes the prediction error for each observation with potentially complex prediction functions.

### What is a novel about the proposed approach?

Instead of a *protection curve* (or estimation of each event as in Price^22^), the “PoDBAY” (Probability of Disease Bayesian Analysis, Supplementary Note 8) framework proposed here uses a *probability of disease curve*, following the original idea of Hobson et al.^19^ This concept enables identification of the slope of the curve and location of 50% protective biomarker value (as does the protection curve), and also a third parameter, the probability that a maximally susceptible (i.e., healthy and seronaive) individual develops a disease, i.e., the maximum probability of disease. (This probability is related to force of infection and placebo incidence rate (“IR”).) We show how to calculate VE and its CI using the probability of disease curve and distributions of the biomarker value in vaccinated and control groups. Using examples based on simulated data we study and compare properties of (i) the proposed PoDBAY method, and (ii) the standard clinical outcome-based VE estimation (using case-counts to estimate proportional risk reduction). (Additional differences with previous work include statistical methods enabling more general relationships between the parameters, as discussed briefly in Results describing influenza virus vaccine analysis and Supplementary Note 10.)

#### What is the benefit? Making decisions using PoDBAY

It is important to clarify that this work cannot replace a formal assessment and validation of immunological CoPs. However, if an immunogenicity biomarker has been sufficiently validated as a CoP using, e.g., some of the formal approaches^8,23–28^ (the first of which was proposed by Prentice)^29^, PoDBAY can be used for assisting important decisions in vaccine development by (i) predicting VE in phase 1 when only immunogenicity data are available, (ii) estimating VE in phase 2 and phase 3 and comparing it to observed VE, (iii) using estimated VE as an alternative metric (rather than arbitrary ratios of geometric mean titers) for lot consistency or immunobridging, or (iv) predicting VE of a new vaccine (for the same pathogen and sufficiently similar mechanism), formulation, or geography, possibly replacing (or at least reducing the size of) some large clinical trials.

#### Outline

In the “Methods” section we introduce a continuous model that infers the probability of disease from the immune response biomarker values of diseased and non-diseased subjects. We then describe how the probability of disease model can be combined with the distributions of observed immune response biomarkers in vaccinated and control groups to estimate VE and its CI. This section also presents simulation methods used to qualify the accuracy and precision of the VE estimate as well as the reliability (calibration) of its CI.

Results of the simulations and the general properties of the method are described in the Results section. The method is illustrated using clinical trial data from influenza virus, zoster virus, and dengue virus vaccines. VE estimates obtained by PoDBAY and by the standard method (using case counts to estimate proportional risk reduction) are compared.

The “Discussion” section highlights the main conclusions and implications for vaccines research and development.

## Results

### Overview of results

PoDBAY relates the probability of disease (“PoD”) to a CoP (generally referred to below as “titer”) via a decreasing sigmoid function. This “PoD curve” is typically created with titer data observed early in clinical trials (e.g., 1-month post vaccination) and is used to estimate VE (based on these data from the same or other trials). Details and caveats are in the “Methods” section.

This section first shows the properties of PoDBAY for simulated data using a range of trial sizes, VEs, and CoP properties. The simulations demonstrate that PoDBAY estimates of VE are typically accurate and more precise than the standard (case-count) estimates of VE, enabling them to contribute to more sensitive and specific development decisions than threshold-based (“absolute”) CoP or case-count-based methods. PoDBAY is then applied to influenza virus, zoster virus, and dengue virus vaccines, and the VE estimates (PoDBAY predictions of VE) are compared with VEs estimated using the standard case-count estimation. For all three vaccine examples, the PoD fit indicates a substantial association between the biomarkers and protection, and efficacy estimated by PoDBAY from relatively little immunogenicity data is predictive of the standard estimate of efficacy, demonstrating how PoDBAY can provide early assessments of VE. In addition, the last example illustrates how PoDBAY can also provide potentially informative VE estimates for (demographic) subgroups of subjects.

### Simulations

In order to illustrate PoDBAY and to test how it behaves in a wide range of conditions, the method is first applied to simulated data. The specific data sets used for the examples based on clinical data are described in the following section.

#### Data generation and parameter estimation

Datasets are generated for a range of different trial scenarios where the underlying truth is set, as described in “Methods”. Generated titers are distributed lognormally. The parameter values provided in Table 1 lead to four different values of true VE:

- 53%, if $et_{50} = 7,{{{{{\mathrm{mean}}}}}}(t_{{{{{{\mathrm{vaccinated}}}}}}}) = 8$ (hereinafter referred to as “simulation scenario A”),
- 66%, if $et_{50} = 6,{{{{{\mathrm{mean}}}}}}(t_{{{{{{\mathrm{vaccinated}}}}}}}) = 8$ (hereinafter referred to as “simulation scenario B”),
- 69%, if $et_{50} = 7,{{{{{\mathrm{mean}}}}}}(t_{{{{{{\mathrm{vaccinated}}}}}}}) = 9$ (hereinafter referred to as “simulation scenario C”),
- 80%, if $et_{50} = 6,{{{{{\mathrm{mean}}}}}}(t_{{{{{{\mathrm{vaccinated}}}}}}}) = 9$ (hereinafter referred to as “simulation scenario D”).

**Table 1:** Parameter values used to set the truth for simulated data.

Parameter	Values Tested
$p_{{{\rm{max}}}}$	0.03
$et_{50}$	6; 7
*γ*	7
Number of subjects, *N*	3000; 30,000
Number of subjects in the vaccinated group	2000; 20,000
Number of subjects in the control group	1000; 10,000
Mean log_2_ titer in the vaccinated group, ${{{{{\mathrm{mean}}}}}}(t_{{{{{{\mathrm{vaccinated}}}}}}})$	8; 9
Mean log_2_ titer in the control group, ${{{{{\mathrm{mean}}}}}}(t_{{{{{{\mathrm{control}}}}}}})$	5
The standard deviation of log_2_ titers in the vaccinated group	2
The standard deviation of log_2_ titers in the control group	2

The absolute values of the parameters can be chosen arbitrarily due to invariance implied by the forms of the equations and distributional assumptions, only the coefficients of variation and different efficacies impact results. The values chosen here represent a range of behaviors likely to be of most interest, as they cover the ranges of behaviors relevant to efficacies above 50% and to the examples below using actual clinical trial data (described below for influenza virus, zoster virus, and dengue virus vaccine analysis).

PoD curve and VE estimation accuracy are evaluated as described in “Methods”. One thousand trials are simulated, and estimated values are compared to true parameters underlying generated data.

Parameter estimates from PoDBAY (Supplementary Tables 1 and 2, and Supplementary Note 5) demonstrate that PoDBAY can appropriately estimate the PoD curve in all these scenarios. High values of *γ* cause the PoD curve to approach the traditional PoD curve used with a CoP: the step-function underlying the threshold-based approach in which subjects above $et_{50}$ are completely protected, and those below it are not at all protected. Supplementary Tables 1 and 2 and the results below from simulations (and those from clinical data examples in the Results section on influenza virus, zoster virus, and dengue virus vaccine analysis) show that we can determine the smooth curves even with relatively little data. The resulting PoDBAY estimates improve substantially VE-based decision-making (such as “go/no-go” to the next development phase) by improving VE estimation precision over that of threshold-based CoP VE estimates (data not shown), just as the CoP-based VE using PoDBAY (hereinafter referred to as “CoP-based VE”) improves it over case-count methods (below).

#### Accuracy of VE point estimation

Supplementary Figure 1 and Supplementary Tables 1 and 2 show consistency in accuracy of the PoD curve estimates across the four different simulation scenarios. True values of the PoD curve parameters fall into corresponding interquartile ranges and are close to the estimated median values. Moreover, with an increasing number of subjects in a simulated trial, increasing accuracy and decreasing variance of the estimated parameters are observed.

**Fig. 1:**
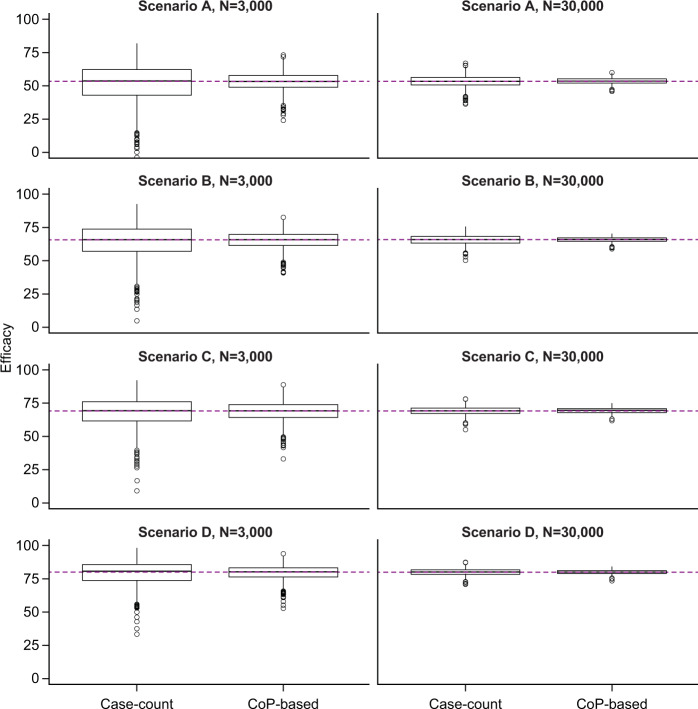
Accuracy of VE estimation. Number of subjects in the simulated trial: *N* = 3000 (left); *N* = 30,000 (right). Numerical results are provided in Supplementary Tables 3 and 4. Interpretation of box plot: center line, median; box limits, upper and lower quartiles; whiskers, 1.5× interquartile range; points, outliers. The common horizontal line represents the true VE used in the simulation.

Figure 1 and Supplementary Tables 3 and 4 show that medians of both case-count and CoP-based VE estimates are close to the true VE in all simulation scenarios (“unbiased estimates”). The CoP-based VE estimates have narrower interquartile ranges and lower variances compared to the case-count VE estimates. The difference between the two methods is greater when there are fewer subjects in a simulated trial.

The value of parameter $p_{{{\rm{max}}}}$ is a function of the IR of the disease in the control population as shown in Eq. (1).

1 $$p_{max} = {{{{{\mathrm{IR}}}}}}_{{{{{{\mathrm{control}}}}}}}\frac{1}{{{{{{{\mathrm{AUC}}}}}}_{{{{{{\mathrm{control}}}}}}}}},$$

where

$${{{{{\mathrm{AUC}}}}}}_{{{{{{\mathrm{control}}}}}}} = \mathop {\int }\nolimits P\left( {{{{{{\mathrm{disease|}}}}}}t_{{{{{{\mathrm{control}}}}}}}} \right) \cdot P\left( {t_{{{{{{\mathrm{control}}}}}}}} \right)dt,$$

with

$P\left( {{{{{{\mathrm{disease|}}}}}}t_{{{{{{\mathrm{control}}}}}}}} \right)$ given as $PoD(t)$ in Eq. (3) with parameter value $p_{{{\rm{max}}}} = 1$, and

$P\left( {t_{{{{{{\mathrm{control}}}}}}}} \right)$ representing the estimated probability density function for observed log_2_ titers in the control (placebo) population.

**Fig. 2:**
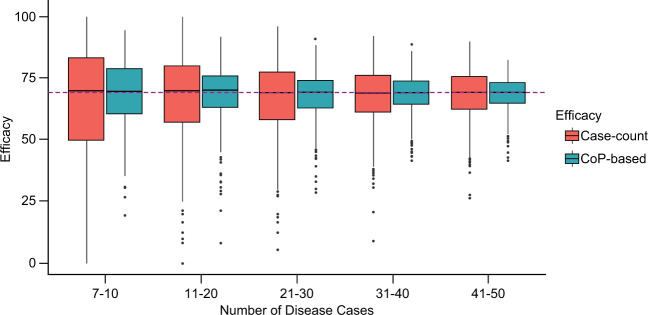
Accuracy of VE estimation when increasing the number of disease cases in a simulated trial. Number of subjects in simulated trial *N* = 3000, simulation scenario C, true VE = 69%, $p_{{{\rm{max}}}}$ = 0.005, 0.01; 0.02; 0.03; 0.04; 0.05. The values of $p_{{{\rm{max}}}}$ were chosen to make a similar number of cases for each group. As expected, some sets with fewer than seven cases do not allow estimation of a PoD curve, and so the lowest category is restricted to seven to 10 cases. More details can be found in Supplementary Note 7. Interpretation of box plot: center line, median; box limits, upper and lower quartiles; whiskers, 1.5× interquartile range; points, outliers. The common horizontal line represents the true VE used in the simulation.

By varying the value of $p_{{{\rm{max}}}}$, the IR and therefore the number of disease cases in a simulated trial can be increased or decreased. To investigate the effect of a number of disease cases in simulated trial on the accuracy of the proposed method, 1000 smaller trials (*N* = 3000) were simulated for each of five variants of simulation scenario C (true VE = 69%) with $p_{{{\rm{max}}}}$ = 0.01; 0.02; 0.03; 0.04; 0.05. As shown in Fig. 2, CoP-based VE has been estimated accurately (any bias is small, as the median is within 1% of true VE) in trials with a number of disease cases higher than seven. As the number of diseased subjects increases, the estimated efficacies are closer to the true VE. The variance of case-count VE estimates is larger than the variance of CoP-based VE estimates, especially in the trials with a low number of disease cases.

#### The precision of VE point estimation

Root mean squared error (“RMSE”, Eq. (8)) and relative root mean squared error (“RRMSE”, Eq. (9)) are calculated for both the CoP-based VE and the case-count VE estimates as described in Methods. Table 2 shows lower values of RMSE and RRMSE for the CoP-based VE estimates compared to the case-count VE estimates, indicating higher precision of the PoDBAY method. The precision of both methods increases with an increasing number of subjects in a simulated trial.

**Table 2:** Precision of the CoP-based VE estimation and the case-count VE estimation based on results from 1000 simulated trials.

Simulation scenario	True VE	Method	RMSE, % (*cf*. Equation (8))		RRMSE, % (*cf*. Equation (9))	
			*N* = 3000	*N* = 30,000	*N* = 3000	*N* = 30,000
A	53%	**CoP-based**	**7.23**	**2.23**	**13.55**	**4.18**
		Case-count	15.36	4.41	28.78	8.27
B	66%	**CoP-based**	**6.46**	**1.90**	**9.82**	**2.88**
		Case-count	13.69	3.79	20.81	5.76
C	69%	**CoP-based**	**7.13**	**2.15**	**10.29**	**3.10**
		Case-count	11.33	3.24	16.37	4.68
D	80%	**CoP-based**	**5.49**	**1.64**	**6.85**	**2.05**
		Case-count	9.26	2.62	11.56	3.27

#### Utility of VE point estimation

The relative performance of the case-count VE estimation and the CoP-based VE estimation is also evaluated using utility as described in Methods. Utility represents an estimate of how often the PoDBAY estimate is closer to the true value of VE than the case-count VE estimate. Because the results are above 50%, the PoDBAY method outperforms the case-count method for all simulation scenarios and both population sizes (Table 3).

**Table 3:** Relative performance of the case-count VE and the CoP-based VE estimation, based on 1000 simulated trials.

Simulation scenario	True VE	Method	Utility, %	
			*N* = 3000	*N* = 30,000
A	53	CoP-based	73.1	71.8
B	66	CoP-based	74.2	70.4
C	69	CoP-based	66.8	64.4
D	80	CoP-based	69.1	68.0

#### Coverage probability of VE CI

To qualify the estimation of the CIs, coverage probability was assessed for the most frequent situation: a trial where immunogenicity is only available for a subset of subjects. (See Methods and Supplementary Note 1 for the algorithm modifications.) Table 4 summarizes the coverage probabilities of the case-count and the CoP-based VE CI estimates. An increasing number of subjects in a simulated trial slightly improves the coverage probability of CoP-based VE CIs. However, even smaller trial (*N* = 3000) simulation results show good coverage probabilities for all simulation scenarios and both methods.

**Table 4:** Coverage probability (%) of case-count VE and CoP-based VE CIs estimation, based on 1000 simulated trials with an immunogenicity subset for a subset of subjects including all disease cases plus 10% of *N*, with *N* the number of subjects in the trial.

Simulation scenario	True VE	Method	*N* = 3000			*N* = 30,000		
			Confidence levels			Confidence levels		
			95%	90%	80%	95%	90%	80%
A	53%	**CoP-based**	**96.6**	**93.1**	**83.0**	**95.3**	**91.7**	**82.8**
		Case-count	94.5	89.6	80.3	94.0	89.5	80.0
B	66%	**CoP-based**	**95.4**	**90.7**	**81.5**	**94.5**	**89.2**	**80.6**
		Case-count	94.7	88.7	77.5	95.3	89.7	79.3
C	69%	**CoP-based**	**96.6**	**91.4**	**81.0**	**96.0**	**90.5**	**78.9**
		Case-count	94.9	90.0	79.9	94.6	89.2	78.1
D	80%	**CoP-based**	**95.5**	**91.3**	**81.8**	**95.1**	**90.8**	**80.5**
		Case-count	95.8	89.7	81.0	95.5	89.8	79.5

#### Width of VE CI

**Fig. 3:**
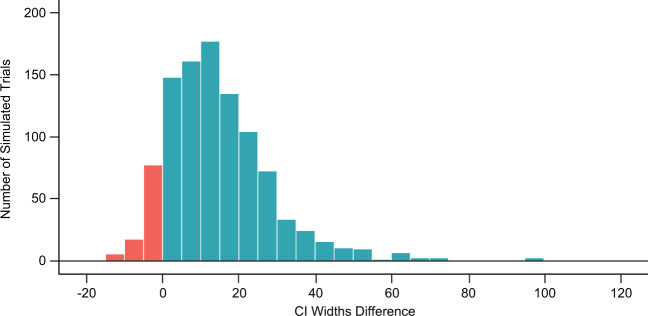
Comparison of widths of the CoP-based VE 95% CIs and the case-count VE 95% CIs. CI widths difference is defined as the width of the CoP-based VE CI subtracted from the width of the case-count VE CI. The number of subjects in simulated trial = 3000, simulation scenario C, true VE = 69%. Red color represents negative CI widths difference, i.e. those in which case-count VE CI width is smaller than that of CoP-based estimate of CI.

Using results of 1000 simulated smaller trials (*N* = 3000), we explore widths of CIs for both methods. The width of a CI is calculated as the lower bound subtracted from the upper bound of the estimated CI. As shown in Fig. 3 and Supplementary Figure 2, the CoP-based VE CIs are narrower than the case-count VE CIs in 90% of simulated trials (blue). The median width of the CoP-based VE CIs is 30, while the median width of the case-count VE CIs is 43.

### Illustrations using clinical trial data

In each of the following three cases, we will outline the data used to create a PoD curve, summarize the immunogenicity data used with that curve to predict the VE, and compare the resulting calculated efficacy to previously published values.

### Influenza vaccines

The adjuvanted trivalent inactivated influenza virus vaccine (“ATIV”, Novartis Vaccines) VE against proven influenza infections was evaluated in a randomized, double-blind, 3-arm, comparative trial that also included non-adjuvanted trivalent inactivated influenza virus vaccine (“TIV”, Novartis Vaccines and GlaxoSmithKline Biologicals), and control in 4707 subjects^30^. Case-count estimate of VE against A/H3N2 in subjects 6–72 months of age for season 2008/2009 was 89% (95% CI: 78–95%) for ATIV and 40% (95% CI: 9–60%) for TIV. Included in this trial was an immunogenicity subset of 777 subjects, documenting their post-vaccination influenza hemagglutination inhibition (“HI”) titers and disease status^31^. (We estimated case-count VE and 95% CI as described in Methods (Standard method: Case-count VE) using numbers of confirmed cases of influenza in TIV vs. control groups, 44/1772 vs. 41/993, reported in Table 1 of Pinheiro et al.^32^)

**Fig. 4:**
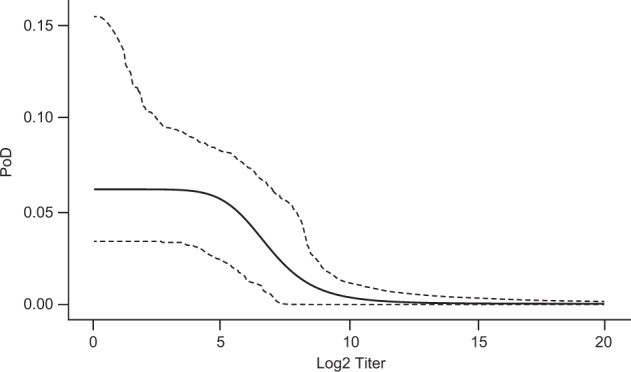
Estimated relationship of influenza HI log_2_ titer and probability of disease. The solid line represents the point estimate of PoD curve; dashed lines represent limits of 95% CI. The estimate includes data on control, ATIV and TIV.

A PoD curve can be estimated (Fig. 4) using individual-level HI antibody titers to A/H3N2 at day 50 post dose 2 in diseased and non-diseased subjects 6–72 months of age for season 2008/2009 from the immunogenicity subset. The dataset consists of titers of all diseased (22 subjects), and all non-diseased (755 subjects) in the immunogenicity sub-study.

The CoP-based VE estimate for ATIV is 85% (95% CIs: 63–97%), based on the estimated PoD curve and influenza HI titers at day 50 post dose 2 in the ATIV group and control group. The CoP-based VE estimate for TIV is 37% (95% CIs: 21–60%), based on the estimated PoD curve and influenza HI titers at day 50 post dose 2 in TIV and control groups.

Black et al. used an immunogenicity dataset^30,31^ to evaluate HI titer in children as a CoP. In this analysis, the compliance with the Prentice criteria is verified, and subsequently, an H3N2 antibody titer (day 50) protection curve (probability of protection curve, “PoP curve”) is estimated and used for the calculation of H3N2 antibody titer levels associated with clinical protection rates of 50%, 60%, 70%, 80%, and 90%. To compare these published results with our results, the PoD curve estimated in this section describing the influenza vaccine was converted to a protection curve in analogy to the work by Dunning^16^ as described in Eq. (2).

2 $${{{\rm{PoP}}}}\left( t \right) = 1 - \frac{{{{{\rm{PoD}}}}\left( t \right)}}{{p_{{{\rm{max}}}}}},$$

where

${{{\rm{PoD}}}}\left( t \right)$ represents the probability of disease curve as given in Eq. (3), and $p_{{{\rm{max}}}}$ represents the probability of disease when log titer ≤ 0.

**Fig. 5:**
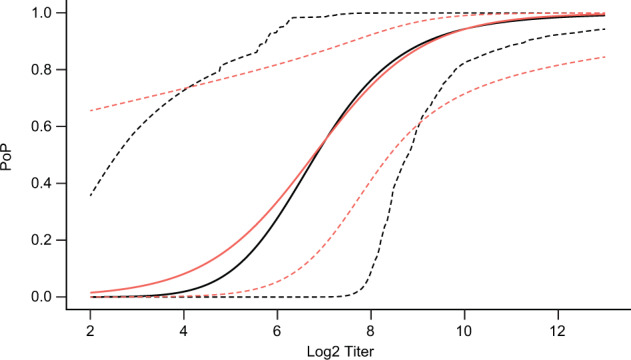
Estimated relationship of influenza HI log_2_ titer to probability of protection. Estimated relationship of influenza HI log_2_ titer to probability of protection with PoDBAY method (black; the solid line represents the point estimate of PoP curve; dashed lines represent limits of 95% CI) and Dunning’s method (red; the solid line represents the point estimate of PoP curve; dashed line represents 95% CI)^16,31^.

Both estimates of the protection curve are provided in Fig. 5. Despite the different mathematical functions used for protection curve estimation, the function values at titer values corresponding to 50% protection rate and higher are comparable (see Fig. 5). Therefore, children’s protective levels of influenza H3N2 antibody titer estimated by our framework (Supplementary Table 5) are very similar to those obtained by Dunning’s method along with the implied clinical and decision-making considerations. In addition to differences induced by the different functional forms for the PoD curve, the difference in CIs is mostly due to PoDBAY including between-trial variability, as well as the non-parametric bootstrap method for calculating CIs (instead of assuming a multivariate normal distribution of parameter estimates). In contrast, Dunning’s method^16^ includes only the parameter uncertainty and uses the multivariate normal assumption via the variance–covariance matrix.

The titer threshold value of 110 established by Black et al.^31^ for, e.g., 50% protection is very close to that estimated here (113). However, the resulting estimates of VE and its CI can be quite different between the PoDBAY and threshold-based approaches to CoP-based VE estimation. For example, if a vaccine yields titers slightly above the 50% protection threshold for 90% of the population, and just below the 50% level for others, the threshold-based method will give a substantial overestimate of VE = 90%, whereas the PoDBAY approach (and Dunning’s) would give a more accurate estimate close to VE = 50%.

In addition, estimates of VE for ATIV and TIV are within 5% of the standard case-count VE estimate (Table 6). CIs of CoP-based VE estimates contain the case-count efficacies for both ATIV and TIV. Similarly, the point estimates of CoP-based VE fall within the CIs of case-count VE. This comparison between estimates of VE and corresponding CIs provides support to the use of PoDBAY for pediatric influenza vaccines, as well as additional support to the influenza HI titer being correlated with protection in children.

This example of inactivated influenza virus vaccine also illustrates one of the most important advantages of CoP-based VE estimation. Even though the case-count VE estimation is based on a trial^30^ six times larger than the immunogenicity sub-study^31^ used for CoP-based VE estimation (4707 vs. 777, as above), the CIs of ATIV VE is for both case-count estimate and CoP-based estimate very similar, and, in the case of TIV VE, the CoP-based VE CI is even narrower than the case-count VE CI (Table 6). This illustrates how earlier data from smaller data sets can be leveraged to accelerate development.

### Zoster vaccine

Zostavax (Merck Sharp & Dohme Corp., a subsidiary of Merck & Co., Inc., Kenilworth, NJ, USA) is one of two currently available vaccines for the prevention of herpes zoster (shingles) which is caused by the reactivation of latent varicella-zoster virus (“VZV”). The phase 3 Shingles Prevention Study (“SPS”)^33^ involved 38,543 subjects and showed that Zostavax had a case-count estimated VE of 51.3% (95% CI: 44.2–57.6%) (Table 2)^34^. An immunogenicity sub-study of SPS that enrolled 1,395 subjects is described in detail by Levin et al.^35^ It has been previously shown that fold rise in antibody titers is a predictor (non-mechanistic, relative CoP) for Zostavax VE under the causal-association paradigm (principal stratification)^36^, effectively using a VE curve approach (which does not require a test for conditional independence relative to vaccination status).

The immunogenicity data consist of a fold rise in titers of all evaluable diseased (32) and non-diseased (1296) subjects in the immunogenicity sub-study (only data from the subjects in the immunogenicity sub-study were used in estimating the PoD curve). The random sampling of fold rise values of non-diseased subjects for determining PoD curve point estimate as described in Supplementary Note 1 is performed accounting for the total number of person-years at risk (3594).

We estimate the PoD curve using individual-level fold rise in VZV antibody titers of diseased and non-diseased subjects in the vaccinated group measured at baseline and week 6 post vaccination by glycoprotein ELISA in the immunogenicity sub-study of SPS. We use values from vaccinated patients as-is: because the control group has (by definition) no change of assay, any measured change is the result of assay drift (or pathogen exposure). Thus, the impact of assay noise (drift) in the prediction is reduced by assuming all values of fold rise in titers of diseased and non-diseased placebo subjects are replaced with the value 1 (log_2_ fold rise equal to 0). (The assay values drifted significantly higher than 1 in the control group, thus inappropriately decreasing the expected control IR and estimated VE.)

**Fig. 6:**
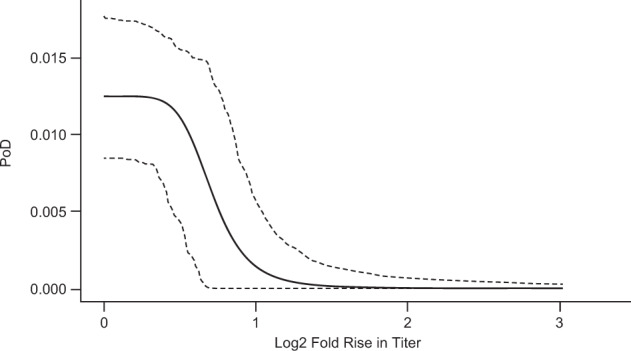
Estimated relationship of log_2_ fold rise in VZV antibody titers post vaccination with Zostavax to probability of disease. The solid line represents the point estimate of PoD curve; dashed lines represent limits of 95% CI.

To obtain CoP-based VE, the estimated PoD curve (shown in Fig. 6) and fold rise in VZV antibody titers in the vaccinated group measured at baseline and week 6 post vaccination are used for the calculation of the numerator of the fraction in Eq. (6). As the log_2_ fold rise in antibody titers is, under our assumption, equal to 0 for all subjects in the control group, the denominator of the fraction is assigned the value of estimated $p_{{{\rm{max}}}}$. The CoP-based VE is 50.5% (95% CI: 40.6–61.0%). While this VE estimate was produced using a fold rise in antibody titers as the predictor, the use of absolute titers produces similar results (data not shown).

The point estimate of the CoP-based VE, 50.5% (95% CI: 40.6–61.0%), is nearly identical to the case-count estimate of 51.3% (95% CI: 44.2–57.6%)^34^, and the CI of Zostavax CoP-based VE is not much wider than the case-count VE CI, despite being based on data from 29 times fewer subjects (1328 vs. 38,501) and 32 times fewer person-years at risk (case-count VE estimation uses 115,939 person-years at risk in the whole SPS study, CoP-based VE estimation uses 3594 person-years at risk in the immunogenicity sub-study, Table 6).

While the VE of the Zostavax vaccine may decrease over time^34^, VZV antibody titers measured by glycoprotein ELISA assay have been shown to be relatively constant^35^. This is an example of how an assay predicting protection can be correlated to (or predictive of) the mechanism of protection when measured at an early time point, while not being correlated with the strength and durability of immune memory and durability of protection. The PoDBAY framework cannot capture or predict the durability of protection in this situation (using VZV antibody titers).

### Dengue vaccine

CYD-TDV is a recombinant, live, attenuated, tetravalent dengue vaccine. In a phase 2b study conducted in Thailand^37,38^, 4002 subjects were assigned to vaccine or control. CYD-TDV is immunogenic and protective in subjects who had previously suffered a dengue infection, but less immunogenic in subjects who are dengue seronegative, as observed in phase 3 trials^39–41^. (Immunogenicity subset size, case-count VE estimates, and other information are shown in Table 6.) We estimate PoD curves for serotype 1 (“DENV1”) and serotype 2 (“DENV2”) using data for the 19 cases caused by DENV1 and the 48 caused by DENV2. This allows CoP-based estimation of overall VE, as well as estimation specifically for the seronegative (or seropositive) populations. This work does not report a new overall VE against DENV1 or DENV2: it will show that the phase 2b data^37,38^ predict (using PoDBAY) VE consistent with the observed phase 3 VE results^39,41^. Because there was a considerably lower observed prevalence of serotype 3 (“DENV3”) and serotype 4 (“DENV4”)^38^, there were too few cases of these serotypes to allow PoD estimation for them. (The low prevalence is likely to have been compounded by higher VE against DENV3 and DENV4^39,41^.)

**Fig. 7:**
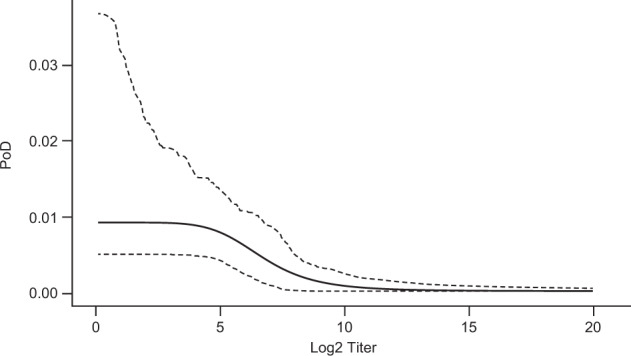
Estimated relationship of DENV1 antibody log_2_ titer to probability of disease. The solid line represents the point estimate of PoD curve; dashed lines represent limits of 95% CI.

**Fig. 8:**
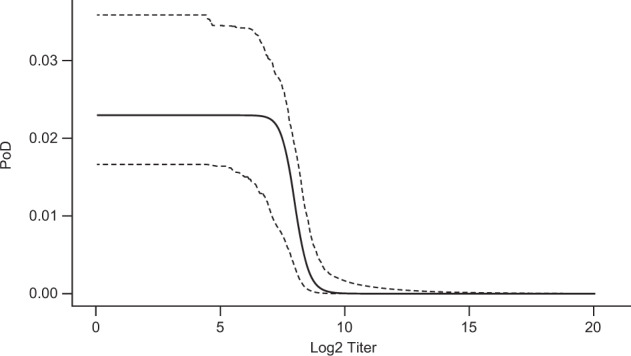
Estimated relationship of DENV2 antibody log_2_ titer to probability of disease. The solid line represents the point estimate of PoD curve; dashed lines represent limits of 95% CI.

For each of DENV1 and DENV2, individual PRNT_50_ antibody titers 28 days post dose 3 for all diseased subjects^37^ and for an immunogenicity subset of non-diseased subjects^38^ are used for PoD curve estimation (Figs. 7 and 8) using the extended method described in Supplementary Note 1.

For DENV1, the CI of the PoD curve and the CI of its $et_{50}$ are considerably larger than for DENV2. This is due to the lower number of disease cases for DENV1 (see above). Titer levels associated with clinical protection rates of 50% against DENV1 and DENV2 are estimated to be 98 and 246 ($2^{et_{50}}$, see Table 5) suggesting different protective levels for different serotypes of dengue virus. However, because the CI of $et_{50}$ for DENV1 is so wide and contains the point estimate of $et_{50}$ for DENV2, such a claim is inconclusive.

**Table 5:** Results of PoD curve estimation for real vaccines.

	PoD curve parameter	Parameter estimate	95% CI
Influenza virus	$p_{{{\rm{max}}}}$	0.063	0.034–0.155
	*γ*	7.379	2.292–45.951
	$et_{50}$	6.824	2.567–8.784
Zoster	$p_{{{\rm{max}}}}$	0.012	0.008–0.018
	*γ*	5.994	2.070–52.948
	$et_{50}$	0.706	0.382–0.991
Dengue, serotype 1	$p_{{{\rm{max}}}}$	0.009	0.005–0.037
	*γ*	6.078	2.034–32.862
	$et_{50}$	6.616	2.183–8.575
Dengue, serotype 2	$p_{{{\rm{max}}}}$	0.023	0.017–0.036
	*γ*	28.655	6.344–36.994
	$et_{50}$	7.945	6.546–8.387

The CoP-based VE is estimated for DENV1 and DENV2 using the corresponding PoD curve estimate and corresponding DENV antibody titers measured 28 days post dose 3 in vaccinated and control groups. The estimates of CoP-based VE are 40% (95% CI: 22–61%) and 40% (95% CI: 26–57%), for DENV1 and DENV2, respectively.

Estimated DENV1 and DENV2 PoD curves using data from phase 2b Thailand study are further used to predict CYD-TDV VE using the titers observed in two phase 3 studies conducted in Asia^39^ and Latin America^41^. The numbers of subjects enrolled, case-count VE, immunogenicity subset sizes, and predicted CoP-based VE in Asia and Latin America studies are summarized in Table 6.

**Table 6:** Results of CoP-based VE estimation for real vaccines: estimated DENV1 and DENV2 PoD curves based on data from the phase 2b Thailand study are used to predict CYD-TDV VE using the titers observed in the phase 3 studies conducted in Asia and Latin America.

		Vaccinated group				Control group				VE	
		N*	Disease cases	Immunogenicity subset		N*	Disease cases	Immunogenicity subset		Case-count	CoP-based
				n*	GMT (95% CI)			n*	GMT (95% CI)	% (95% CI)	% (95% CI)
Influenza ATIV		1937	9	311	746 (661–843)	993	41	153	12 (9–15)	89 (78–95)	85 (63–97)
Influenza TIV		1772	44	313	92 (74–115)	993	41	153	12 (9–15)	40 (9–60)	37 (21–60)
Zoster		58,203	315	1792	1.68 (1.63–1.75)**	57,736	642	1,802	–	51 (44–58)	51 (41–61)
Dengue Thailand	Serotype 1	2536	9	95	146 (99–217)	1251	10	49	24 (14–41)	56 (−22 to 84)	40 (22–61)
	Serotype 2	2510	31	95	310 (224–431)	1250	17	49	52 (27–102)	9 (−75 to 51)	40 (26–57)
Dengue Asia	Serotype 1	6548	51	1,316	166 (150–183)	3210	50	657	47 (39–56)	50 (25–67)	31 (16–53)
	Serotype 2	6561	38	1,314	355 (327–386)	3253	29	657	69 (57–82)	35 (−9 to 61)	42 (34–56)
Dengue Latin America	Serotype 1	12,478	66	1,301	395 (353–441)	6196	66	643	121 (101–145)	50 (29–65)	37 (23–54)
	Serotype 2	12,495	58	1,301	574 (528–624)	6219	50	643	129 (109–152)	42 (14–61)	52 (44–63)

*Number of subjects or number of person-years at risk.

**Geometric mean of fold rise in antibody titers from baseline to week 6.

We predict CoP-based VE for Latin America study in seropositive and seronegative subpopulations for DENV1 and DENV2 using corresponding PoD curve estimates from Thailand 2b study and corresponding DENV antibody titers in seropositive and seronegative subpopulations^41^. Predicted CoP-based VE for DENV1 in the seropositive subpopulation is 10% (95% CI: −19 to 19%) and for DENV1 in the seronegative subpopulation is 27% (95% CI: 7–60%). The apparent reversal (of efficacy for seropositive versus seronegative) can be explained by the large uncertainty (reflected in the large CI) on the PoD curve, which results from the low number of DENV1 disease cases in the Thailand study. Predicted CoP-based VE for DENV2 in the seropositive subpopulation is 49% (95% CI: 35–56%) and in the seronegative subpopulation is 26% (95% CI: 19–54%). Case-count efficacies for seropositive and seronegative subpopulations in the Asia study^39^ were not published for individual serotypes, however, the overall efficacies across serotypes that were published do show substantial differences between the seropositive and seronegative populations in the Asia study:^39^ case-count efficacy combining all four serotypes was 74% (95% CI: 53–86%) in seropositive subjects and 36% (95% CI: −27 to 67%) in seronegative subjects.

While a CoP for DENV has not been yet established, recent advances in research toward its identification as well as remaining challenges were summarized by Katzelnick et al.^42^ and have also been studied by Salje et al.^43^, and by Moodie et al.^44^ These investigations suggest that PRNT_50_ DENV antibody titers measured 28 days post dose 3 are likely to be a relative correlate of protection. According to our results (generated from a smaller data set), these titers are predictive of protection by CYD-TDV for serotype 1 and serotype 2 (and, thus, are a relative CoP at least for those serotypes). Of course, results from CYD-TDV (e.g., parameters for the PoD curve) may not be directly applicable to other dengue vaccines, as even a very useful (predictive) CoP can have PoD parameters that are mechanism dependent. Also, this analysis does not demonstrate that the antibody titers predict the likelihood of disease independent of vaccination status: this could not be assessed because the data^37^ used to generate the PoD curve were not labeled with vaccine status.

In the case of DENV1, point estimates of VE based on PRNT_50_ DENV antibody titers are consistently approximately 10–20 percentage points lower than case-count VE estimates across all analyzed trials, but all point estimates fall within the case-count method’s CI and CIs of CoP-based VE and case-count VE overlap considerably.

DENV2 VE estimates based on antibody titers are ~10 percentage points higher than case-count VE estimates in both phase 3 trials and their CIs overlap substantially. CoP-based VE CI is more than four times narrower than case-count VE CI while being based on the same number of subjects (3760 subjects in the whole phase 2b study, Table 6).

There is a large discrepancy in DENV2 estimated efficacies obtained by the case-count method for the Thailand phase 2b trial and phase 3 trials. In Thailand’s phase 2b study, the case-count method estimated the VE to be 9%. However, when the vaccine was tested in larger phase 3 trials, the case-count method estimated the VE to be between 35 and 45%. This discrepancy in estimated efficacies is not uncommon with the case-count method: the PoDBAY method consistently estimated the DENV2 VE to be approximately 40% for both the phase 2b and the two-phase 3 trials (Table 6).

To understand the likelihood of such a large difference in CoP- versus standard-VE, simulated data were generated using true VE 40% to mimic the properties of the Thailand phase 2b study. One-thousand instances of a phase 2b trial were generated, and the VE was estimated using the case-count method and the PoDBAY method. For roughly 8% of the 1000 simulated trials, the case-count method estimated a VE of less than 9% when in fact the underlying true VE was 40%. Therefore, when conducting a vaccine trial with a small number of subjects, there is a significant risk of a clinically relevant discrepancy between case-count-estimated efficacy and true efficacy.

**Fig. 9:**
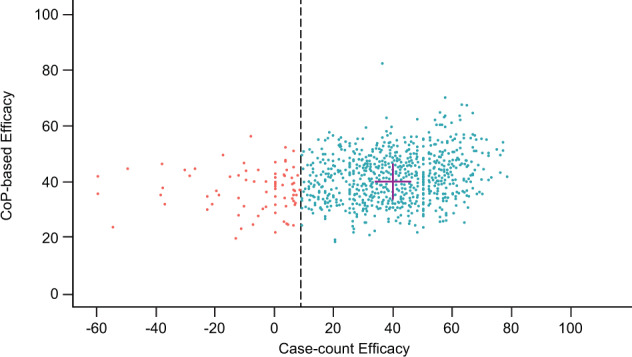
Case-count VE substantially underestimates VE in a substantial portion of trials; PoDBAY eliminates most of these outliers. Point estimates of CoP-based VE and case-count VE in 1000 simulated trials, with true VE set to 40% and a number of subjects *N* *=* 3760. Simulated trials with case-count VE < 9% are shown in red.

Figure 9 shows that for a large proportion of instances where the case-count method estimated very low VE, the PoDBAY method estimated a VE that was closer to the true VE of 40% (as set up in the simulation). This is visible in the vertical spread of the red points (and all points) being smaller than their horizontal spread. This phenomenon suggests that there is a chance that the case-count method could lead to a “no-go” decision after a phase 2b trial, even when the vaccine is efficacious. On the other hand, there is a much smaller chance of making an unwarranted “no-go” decision using the PoDBAY method. Moreover, the CoP-based VE estimate based on the Thailand phase 2b study is closer to the estimated efficacies for the two-phase 3 studies, a reminder (cf. Fig. 4) that the CoP-based VE is more independent of trial size than case-count VE.

For all three dataset examples, the VE estimated by PoDBAY is consistent with the case-count estimate of VE. For influenza virus and zoster, this confirms previous work^30,31,36^ showing that biomarkers used for the estimations are correlated with protection. For dengue, where a CoP has not been established, the consistency between case-count- and CoP-based VE estimates suggests a dengue virus neutralizing antibody titer is likely to be a CoP and encourages further efforts toward its evaluation. The statistical efficiency of the PoDBAY method is evident from the similar or narrower CIs obtained from a much smaller immunogenicity subset of patients in those studies.

## Discussion

### Why can a continuous (“relative”) CoP be better than thresholds (“absolute” CoPs)?

Important decisions in vaccines research and development traditionally require a threshold-based CoP to be identified as a biomarker (subjects with titers above the threshold are considered completely protected). This threshold-based CoP is often not available. However, internal decisions have been facilitated by requiring a geometric mean biomarker value (and, sometimes, lower bound of a CI) to be above a threshold.

Both of these threshold-based decisions are sensitive to outlier effects and their decision criteria may not be directly related to clinical significance. PoDBAY integrates the whole distribution of biomarker values with clinically relevant information, providing a framework to more accurately and precisely estimate VE and its CI. This allows decisions to be made using clinical and public-health-based targets for VE (and CI lower bound). PoDBAY can be applied to analyze both absolute and relative CoPs. As shown in the influenza virus vaccine analysis, PoDBAY can produce substantially different efficacy (and CI) estimates than typical threshold-based absolute CoP estimation. Further, PoDBAY does not require that either titers or PoD curve parameters fit standard distributional assumptions (such as multivariate normality). The importance of considering both the mean and variability of the value was suggested also by Nauta et al.^45^.

### PoDBAY versus case-count?

When applied to simulated data and real trial data, PoDBAY accurately predicted VE and had the significant benefit of providing higher precision VE (narrower CIs) than that of case-count VE (for the same number of subjects). In addition, PoDBAY outperformed the case-count VE estimation in terms of precision as shown (in simulations) by the RMSE, RRMSE, and utility: estimated VE values were closer to the true value in a larger percentage of simulations compared to the case-count method. The higher precision VE (narrower CIs) was obtained while preserving well-calibrated CIs (coverage probability). Of course, if the PoD model is not correct, the method may produce biased results, but additional simulations have shown that VE estimates are relatively insensitive to misspecification of the curve shape (data not shown, see also Dunning^17^).

### What is the benefit? Making “go/no-go” decisions using PoDBAY

One example of the potential impact of these results is the implied improvement in a decision (e.g., on whether to continue development of a vaccine candidate) made by requiring estimated efficacy to be above a given threshold.

Non-clinical immunogenicity (and, sometimes, protection) data often provide the evidence enabling a vaccine candidate to be tested in clinical trials. Adding a translational version of PoDBAY (e.g., using data from animal challenge studies to understand titer levels that provide protection in appropriate model species) is effectively an extra layer of risk reduction. Here, in addition to immunogenicity (often defined as a statistically significant difference between titers of animals receiving control and vaccine) and/or evidence of protection in model species, continued development of the vaccine candidate would also require confidence in the relationship between the titer and protection. (Such confidence should be supported in any use by, for example, a PoD with a negative slope.) This could further justify clinical testing, prioritize among candidates, or prevent testing of a candidate with a low probability of efficacy.

A decision to bring a candidate from phase 1 to phase 2 has sometimes been based on the statistical significance of the difference between control and vaccinated populations, or on potentially arbitrary rules (based, for example, on assay variability) such as at least two- or four-fold increase in geometric mean titer. Here, PoDBAY can provide an additional criterion to support the decision, increasing the likelihood that a good candidate would progress and that a poor candidate would not. Use of PoDBAY for this decision (progression to phase 2) would require more evidence (for the PoD relationship) than for the decision to progress to phase 1: there is a need to ensure that the potential benefit warrants the larger number of subjects and increased resource use.

To make a decision to proceed to phase 3, an even stronger case should be made. This would best be done in a proof of concept phase 2 trial accruing a small number of cases collected to demonstrate the likely efficacy and (again through, e.g., a PoD curve with negative slope) the likely existence of a CoP. As another approach, evidence for a CoP has also been provided using challenge experiments in multiple model species (potentially including humans), and then showing that the PoD relationship can be appropriately translated to clinical application^46–48^. This approach also used summary-level data and model-based meta-analyses to provide evidence of a biomarker’s predictive power. When performing these and other analyses, applying results (especially parameters for the PoD curve) for one vaccine directly to other vaccines for the same pathogen should be done only with the utmost care, as even a very useful CoP can have PoD parameters that are mechanism-dependent.

In order to use a CoP-based VE estimate to justify filing for approval requires strong and sufficiently independent clinical evidence. This would likely be from a previous trial. It would also require sufficient evidence that the CoP is, by itself, sufficiently predictive. The latter evidence could include the conditional independence (of PoD from vaccine status) required by the Prentice criteria^29^, if not additional evidence of robustness to the other factors mentioned as part of the formal assumptions and related statistical considerations^49^. Because demonstration of the Prentice criteria often requires substantially more cases than estimating a PoD curve, it is often possible only in phase 3 and beyond.

Because the precision (and utility metric) of the CoP-based VE is higher than that of case-count, the estimate is more likely to be on the correct side of a decision threshold—whether the correct side is above (a vaccine that should be developed), or below (efficacy is too low, and development should be stopped or candidate should be modified) that threshold. Further, the ability of PoDBAY to estimate VE more precisely (with a narrower CI) can be particularly impactful, as many “go/no-go” decisions rely on the lower limit of a CI.

Simulation results (Supplementary Note 6) show that the decisions using PoDBAY have more power while still controlling the type I (“false positive”) error rate. In addition, when a CoP-based VE estimate can be used, the decision (relying on either a point estimate of VE or the lower limit of VE CI) will in general have higher accuracy—both sensitivity and specificity. This enables better-informed “go/no-go” decisions in vaccines research and development.

Specifically, results of phase 2 trial simulations show that when true VE is 40%, there is a 3× lower chance to make unwarranted “go” decision using PoDBAY than with case-count. (Details of phase 2 and 3 simulations can be found in Supplementary Note 6.) Similarly, when true VE is 60%, there is a 3× lower chance to make unwarranted “no-go” decision when using PoDBAY. Similarly, in phase 3 the chance of an incorrect decision is several-fold higher using case-count than using PoDBAY. The phase 3 trial simulation results also show that there is a smaller chance of making unwarranted false positive “go” decisions with PoDBAY than with case-count, with only a small chance of such an error using either approach.

### When is PoDBAY most reliable?

When there is sufficient evidence that the immune response biomarker is a CoP, PoDBAY is an attractive time- and cost-effective option as it can accurately predict VE on a shorter time scale while using significantly fewer subjects than counting clinical endpoint assessments. Such evidence includes fundamental virology and immunology and should be supported at least by in vitro or (non-clinical) in-vivo evidence of the biomarker being predictive of reductions of symptoms and/or viral load.

The use of the biomarker can then be further substantiated by the strength (“goodness-of-fit”) of the PoD model (e.g. the p-value of non-null parameter values) relating biomarker to clinical case probability, and by the degree to which standard- and CoP-VE are sufficiently close when estimated using different data. Here, as explained above, the definition of “sufficiently close” will depend on the phase of the study, the potential clinical impact of an incorrect decision, etc., and sufficiency can also be supported by evidence that the PoD holds across multiple dose-levels, formulations, or even vaccines. As discussed in Methods (Data collection and assumptions) and Supplementary Note 10, the method *formally* requires assuming that the PoD relationship is independent of other factors (demographic, immunological, or experimental); however, standard techniques provide straightforward extensions to PoDBAY for identifying and quantifying the impact of any such factors.

Furthermore, CoPs and related surrogate marker paradigms are always used with consideration of a risk-benefit tradeoff. Also, immunogenicity markers that are not putative CoPs have been successfully used for applications such as bridging between populations and lots; use in bridging (also between formulations) might not require the highest standards of proof (such as needed for CoP-based filing). Vaccines have even been approved based purely on immunological endpoints that were not CoPs, but for which there was strong mechanistic evidence directly connecting Ab titers to protection^50^.

### Implications for COVID-19 vaccine development

Methods like PoDBAY can help accelerate and economize vaccine development in the current effort against the COVID-19 pandemic, as they can provide information for prioritizing (ranking) candidates both early in development and earlier in a trial. For example, information from trials (including non-clinical and clinical with approved/candidate vaccines or with intravenous immunoglobulin) can be leveraged to estimate a PoD curve. (This requires assuming, for example, that serum neutralizing titers (“SN”) would be sufficiently predictive to at least rank order candidates correctly most of the time.) If several candidates are tested and SN data become available, the distributions of predicted efficacies for the candidates can be compared, and the comparisons used to focus resources on the most promising. In addition, Bayesian adaptive strategies^51^ and master protocols^52^ can also be informed using these techniques, where early stage information on cases and titers may also help define and/or refine the PoD curve, thus enabling immunogenicity information to be used fairly and more confidently to compare potential candidates.

### Future research

Future work should investigate how PoDBAY behaves in several conditions including: with a bacterial vaccine, when a larger data set is available (such as that from the CYD-TDV phase 3 studies^44^), if its principle assumption of the biomarker being a CoP is violated, as well as on extending PoDBAY to multiple predictors and using the PoDBAY framework for testing conditional independence. Systematic analysis of the sensitivity of PoDBAY to PoD curve model misspecification analogous to Dunning^17^ and the use of model selection or model weighting schemes^32,53,54^ could help quantify misspecification-related risks. Such risk analyses also need to consider identification of potential PoD factors other than titer (e.g., conditional independence tests) and their potential for confounding VE estimation, as well as the potential impact of other factors and the considerations related to multiple-trial settings.

## Methods

### Data collection and assumptions

In a typical clinical trial, subjects are randomly assigned to receive one or more dose(s) of vaccine or placebo, and the occurrence (or non-occurrence) of disease in each subject is recorded over a subsequent surveillance period.

Serum samples are collected in all subjects during one common time window (e.g., 40 ± 10 days) after vaccination (and, if needed, at baseline, i.e., before vaccination) and assayed. (In the frequent case that only a subset of subjects is assayed for biomarker values, the data must be carefully upsampled to properly represent actual IRs. Supplementary Note 1 describes this extension of the method in more detail.) Without loss of generality, we refer to the assay (or biomarker) as “titer,” since neutralizing antibody titer is often used.

The implementation of the method described here assumes that the surveillance period is equal for all subjects, although in some situations this can be relaxed. We also assume that titer is a CoP, meaning that the titer level after vaccination is associated with protective immunity regardless of treatment group (in other words, that the vaccine’s effect on the disease goes completely through the titer; as discussed further in the Discussion section, for practical use this assumption only needs to hold approximately). This work does not consider the potential influence of other demographic, immunological, or experimental factors (sometimes termed “covariates”); Supplementary Note 10 discusses how straightforward extensions for identifying and quantifying any such influence can be made using standard techniques that are widely used even in regulatory decision-making^55^.

All software was written in and produced using R statistical software version 3.5.2.

### Proposed PoDBAY method

#### Estimation of probability of disease curve and its CIs

**Fig. 10:**
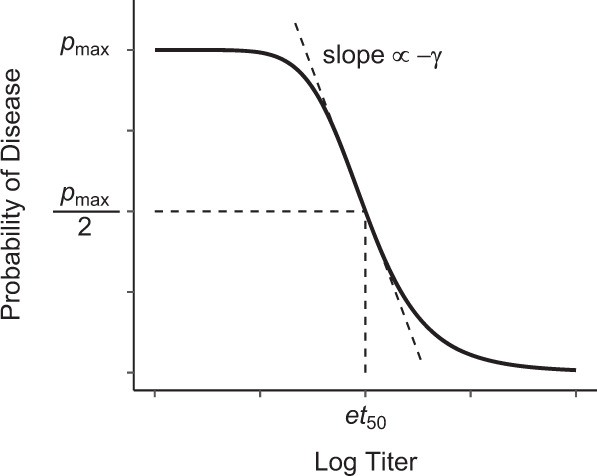
The PoD curve and its parameters. Sigmoid relationship between log titer and probability of disease (PoD curve).

The PoD is modeled as a decreasing sigmoid function of the log titer (log-transformed titer), as illustrated in Fig. 10 and Eq. (3), and can be performed on blinded data (i.e., requires information about subject disease status and immunogenicity biomarker values, but not on the treatment). The PoD can be thought of as the expected IR per subject for a given observation period for a large group of subjects with titer *t*: equivalently it is the probability a vaccine recipient with antibody titer *t* would have the disease during the study period (and with “disease” defined using the relevant study endpoint). While the amplitude of this function (represented below by $p_{{{\rm{max}}}}$) can vary with time (due to, e.g., seasonally varying force of infection), if placebo and vaccine arms of a trial are simultaneous (as is typical), such variation cancels out in all efficacy calculations that follow and thus can be safely ignored. Similarly, geographic variation can be ignored if the arms are co-located.

3 $$PoD\left( t \right) = \left\{ {\begin{array}{*{20}{l}} {p_{{{\rm{max}}}}\qquad\qquad{{{{{\mathrm{if}}}}}}\;t \,\le\, 0} \hfill \\ {p_{{{\rm{max}}}} \cdot \frac{{\left( {\frac{{et_{50}}}{t}} \right)^\gamma }}{{1 + \left( {\frac{{et_{50}}}{t}} \right)^\gamma }}\,{{{{{\mathrm{if}}}}}}\;t \, > \, 0,} \hfill \end{array}} \right.$$

where *t* represents log titer value, $p_{{{\rm{max}}}}$ represents the maximum probability of disease when log titer ≤ 0, $et_{50}$ represents the log titer value for which the probability of disease is half of $p_{{{\rm{max}}}}$, and *γ* represents the slope of the curve.

The logarithm of titer is used here because raw titer values are typically lognormally distributed^56^, and titers are often represented by their log_2_ values. The PoD curve could also use titers or fold rise in titers from baseline rather than log titers. (The cutoff of log titer ≤ 0 is an arbitrary choice for computational convenience and does not impact the method: since titers are generally defined as dilution factors, they are always at least 1, so that the log titer > 0. In the event a different assay form is used, a simple change of units can be used to ensure log titer > 0. The cutoff could be also generalized to log titer ≤ *c*, probably as a prespecified user input based on assay properties, but also potentially fit with data if supported by data and the application.)

Three models (piece-wise linear, logistic, and piece-wise exponential) were used to simulate data that were subsequently fit by the three-parameter sigmoid model defined in Eq. (3). The results (data not shown) indicated that the proposed model is flexible and can fit the data well, as the assumption of the specific three-parameter sigmoid in Eq. (3) had relatively little effect on the precision and accuracy of VE estimation. Due to this finding and its agreement with similar work by other authors (e.g., Dunning^17^), this work did not prioritize model selection or weighted model combination mechanisms and related goodness-of-fit evaluation^32,53,54^.

To estimate parameters of the PoD curve, a maximum likelihood estimation (“MLE”) method is used. The basic principle is to start with the assumption that Eq. (3) holds, and to first find the parameter values that make it most likely (among all choices of parameters) that the observed data would occur (the “maximum likelihood estimate,” or MLE). This likelihood, *L*, is calculated, using the PoD curve, Eq. (3), and the disease status of all subjects, with Eq. (4).

4 $${{{{{\mathrm{Log}}}}}}L = \mathop {\sum }\limits_{{{{{{\mathrm{diseased}}}}}}}^{} {{{{{\mathrm{log}}}}}}\left( {{{{\rm{PoD}}}}\left( t \right)} \right) + \mathop {\sum }\limits_{{{{{{\mathrm{non - diseased}}}}}}}^{} {{{{{\mathrm{log}}}}}}\left( {1 - {{{\rm{PoD}}}}(t)} \right),$$

Here, ${{{\rm{PoD}}}}(t)$ is given in Eq. (3), and the sum over diseased subjects uses the values of *t* for the subjects who had the disease during the observation period, and analogously for the other sum and non-diseased subjects. Eq. (4) is analogous to that used commonly for logistic regression and has the intuitive property that, if all the PoD values of the first (diseased) term are as high as possible (close to $p_{{{\rm{max}}}}$, such as when all the titers are much lower than $et_{50}$), and all the PoD values of the second (non-diseased) term are as low as possible (as when the titers are much higher than $et_{50}$), then *L* will be as large as possible. The optimal parameters are those that maximize the log-likelihood (5).

5 $$p_{{{\rm{max}}}}^ \ast ,et_{50}^ \ast ,\gamma ^ \ast = \mathop {{{{{{{\mathrm{argmax}}}}}}}}\limits_{p_{{{\rm{max}}}},\,et_{50},\gamma } \left( {\log L} \right)$$

The L-BFGS-B algorithm^57^ is used for the optimization. This method (MLE estimate of the parameters in Eq. (3)) is very similar to logistic regression but has an extra parameter representing the maximum probability of disease (for those with lowest titers), and a slightly different shape for the sigmoidal curve. (Dunning’s original work^16^ used MLE to get the two parameters of inverse logit function *α*, *β* and the parameter (*λ*) analogous to $p_{{{\rm{max}}}}$.)

The parameter values in Eq. (5) give the “point” or “central” estimate of the PoD curve. The CI around the PoD curve is estimated using a non-parametric bootstrap, as explained in Supplementary Note 2.

#### Estimation of VE and its CI

Based on the estimated PoD curve and the estimated probability density function for observed log titers, the expected rate of disease in the vaccinated and control groups can be calculated and used for VE estimation as shown in Eq. (6).

6 $${\rm CoP \hbox{-}based \; VE} \;=\; 1 - \frac{{E\left[ {{{\rm{PoD}}}_{{{{{{\mathrm{vaccinated}}}}}}}} \right]}}{{E\left[ {{{\rm{PoD}}}_{{{{{{\mathrm{control}}}}}}}} \right]}},$$

where

$$E\left[ {{{\rm{PoD}}}_{{{{{{\mathrm{vaccinated}}}}}}}} \right] = \mathop {\int }\nolimits P\left( {{{{{{\mathrm{disease|}}}}}}t_{{{{{{\mathrm{vaccinated}}}}}}}} \right) \cdot P\left( {t_{{{{{{\mathrm{vaccinated}}}}}}}} \right)dt,$$

$$E\left[ {{{\rm{PoD}}}_{{{{{{\mathrm{control}}}}}}}} \right] = \mathop {\int }\nolimits P\left( {{{{{{\mathrm{disease|}}}}}}t_{{{{{{\mathrm{control}}}}}}}} \right) \cdot P\left( {t_{{{{{{\mathrm{control}}}}}}}} \right)dt,$$

where $E\left[ {{{{\rm{PoD}}}}_x} \right]$ represents expected rate of disease in group *x* (vaccinated or control), $P\left( {{{{{{\mathrm{disease|}}}}}}t_x} \right) = {{{\rm{PoD}}}}\left( t \right)$ (Eq. (3)), with titers *t* for subjects in group *x*, and $P\left( {t_x} \right)$ represents estimated probability density function for observed log titers in group *x*.

The above estimate of VE is a point estimate based on CoP and is obtained using the central estimate of the PoD curve defined in Methods (Estimation of probability of disease curve and its associated CIs). The calculation of the CI of CoP-based VE is described in Supplementary Note 3.

### Standard method: case-count VE

VE is routinely estimated using case counts to estimate the reduction in IR in vaccinated relative to control subjects as described in Eq. (7).

7 $${{{{{\mathrm{case\hbox{-}count}}}}}}\;{{{{{\mathrm{VE}}}}}} = 1 - \frac{{{{{{{\mathrm{IR}}}}}}_{{{{{{\mathrm{vaccinated}}}}}}}}}{{{{{{{\mathrm{IR}}}}}}_{{{{{{\mathrm{control}}}}}}}}},$$

where

$${{{{{\mathrm{IR}}}}}} = \frac{{{{{{{\mathrm{number}}}}}}\;{{{{{\mathrm{of}}}}}}\;{{{{{\mathrm{diseased}}}}}}\;{{{{{\mathrm{subjects}}}}}}}}{{{{{{{\mathrm{number}}}}}}\;{{{{{\mathrm{of}}}}}}\;{{{{{\mathrm{all}}}}}}\;{{{{{\mathrm{subjects}}}}}}}}.$$

To obtain the CI of VE estimated in Eq. (7), a 2 by 2 contingency table is constructed for the number of subjects in each of the combinations of vaccinated or control and diseased or non-diseased, and the Wald method^58^ for calculating the CI of the relative risk can be used. In specific trials, other estimates of risk reduction (and its CI) can be used to account for person-years at risk, and covariates such as location, ethnicity, and age. The principles involved and expected results (relative to PoDBAY) will still be represented appropriately by the methods used here.

### Clinical trial simulations to compare proposed method and case-count VE (standard) method

**Fig. 11:**
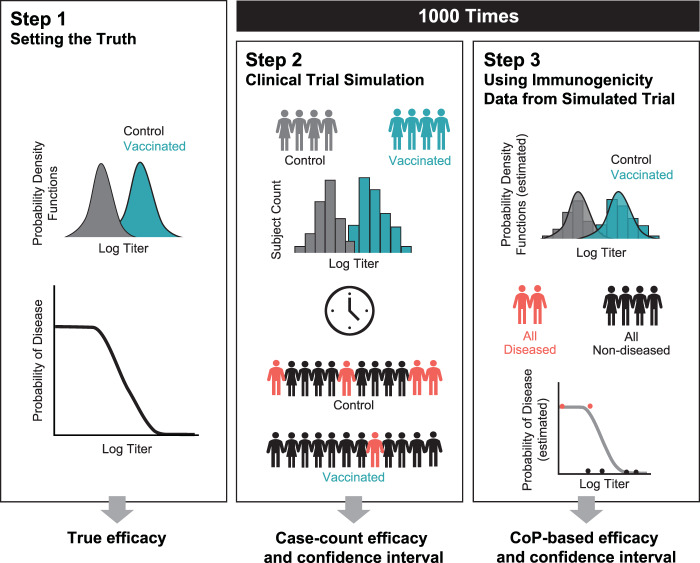
Clinical trial simulation workflow. Steps used to assess accuracy and precision of the proposed method.

To assess the accuracy and precision of the proposed method, its performance relative to the case-count method, and the coverage probability of the CI estimation, three steps are followed (see Fig. 11):

Step 1: Assumed true values are assigned to PoD curve parameters ($p_{{{\rm{max}}}}$, $et_{50}$, *γ*) and log titer distribution parameters (mean, sd, assuming normal distribution) for the vaccinated and control group.

Step 2: Log titer data are generated for the whole vaccinated and control population using random sampling from true distributions. Disease status is assigned to each subject randomly using the probability of disease defined by the true PoD curve. Case-count VE and its CI are estimated as described in Methods (standard method: case-count VE).

Step 3: Individual titers of all diseased and all non-diseased subjects are used to estimate PoD curve parameters and their CIs, as described in Methods (Estimation of probability of disease curve and its CIs). The probability density function parameters for the vaccinated (resp. control) group are estimated using the titers of vaccinated (resp. control) subjects. CoP-based VE and its CI are estimated, as described in Methods (estimation of VE and its CI).

Steps 2 and 3 are repeated 1000 times to yield 1000 estimates of case-count VE with corresponding CIs, 1000 PoD curve parameter combinations, and 1000 estimates of CoP-based VE with corresponding CIs. (Increasing the number of replicates did not substantially change the results—as expected for such an analysis of 1000 Bernoulli trials.) Point estimates of case-count VE and CoP-based VE are compared to the true VE to evaluate the accuracy of both methods. Point estimates of PoD curve parameters are compared to the true values of PoD curve parameters to evaluate the accuracy of PoD curve estimation. Root mean squared error (Eq. (8)) and relative root mean squared error (Eq. (9)) of case-count VE estimates and CoP-based VE estimates are calculated to evaluate precision of both methods. Because efficacy is always between 0 and 100%, RMSE enables a useful absolute comparison between models whose errors are measured in units of efficacy.

8 $${{{{{\mathrm{RMSE = }}}}}}\sqrt {\frac{{\mathop {\sum }\nolimits_{i = 1}^{N_{{{\rm{sim}}}}} \left( {{{{{{\mathrm{VE}}}}}}_{{{\rm{true}}}} - {{{{{\mathrm{VE}}}}}}_{{{{\rm{estimated}}}},i}} \right)^2}}{{N_{{{\rm{sim}}}}}}} ,$$

9 $${{{{{\mathrm{RRMSE = }}}}}}\sqrt {\frac{{\mathop {\sum }\nolimits_{i = 1}^{N_{{{\rm{sim}}}}} \left( {\frac{{{{{{{\mathrm{VE}}}}}}_{{{\rm{true}}}} - {{{{{\mathrm{VE}}}}}}_{{{{\rm{estimated}}}},i}}}{{{{{{{\mathrm{VE}}}}}}_{{{\rm{true}}}}}}} \right)^2}}{{N_{{{\rm{sim}}}}}}} = \frac{{{{{{{\mathrm{RMSE}}}}}}}}{{\mathrm{VE}_{{{\rm{true}}}}}},$$

where *N*_sim_ is the number of simulations performed.

One measure of the relative performance of two estimation methods is utility, defined here as a percentage of cases when CoP-based VE estimate is closer to the true value of VE than the case-count VE estimate. To understand the reliability (accuracy) of the CI boundaries, the “coverage probability” of case-count VE CI estimates and CoP-based VE CI estimates are calculated for confidence levels of 80%, 90%, and 95%. The coverage is the percent of simulations in which the true efficacy falls within the CI, which should be (for example) 80% for the 80% CI. Widths of case-count VE CI and CoP-based VE CI are compared.

### Reporting summary

Further information on research design is available in the Nature Research Reporting Summary linked to this article.

## Supplementary information

Supplementary material (clean)

Reporting Summary

## Data Availability

All data that support the findings in this work are available publicly from the cited references except for the subject-level data used in the zoster virus vaccine analysis. The data that support the findings in that section will be made available according to the data sharing policy of Merck Sharp & Dohme Corp., a subsidiary of Merck & Co., Inc., Kenilworth, NJ, the USA, which is available at http://engagezone.msd.com/ds_documentation.php. Requests for access to the clinical study data can be submitted through the Engage Zone site or via email to dataaccess@merck.com.

## Code availability

A package implementing the PoDBAY algorithm is available in the Comprehensive R Archive Network at https://cran.r-project.org/web/packages/PoDBAY/index.html.
