# Supplementary material for: A method to estimate probability of disease and vaccine efficacy from clinical trial immunogenicity data
Source: NPJ Vaccines. 2021 Nov 4;6:133. doi: 10.1038/s41541-021-00377-6 (PMC8568947; doi:10.1038/s41541-021-00377-6)
Supplement: Supplementary file 1 — Supplementary material (clean) [file 41541_2021_377_MOESM1_ESM.pdf]

Supplementary Material

Supplementary Note 1: Estimation of Parameters When a Subset of Subjects is Sampled for Immunogenicity

In the frequent case when serum samples at baseline and after vaccination are collected and assayed only in a subset of subjects (hereinafter referred to as "immunogenicity subset") and the assay value of titer is obtained also for all disease cases at the same timepoints, the method for PoD curve estimation described in Methods (Estimation of probability of disease curves and its confidence intervals) can be extended as follows.

To obtain a reliable MLE estimate of the PoD curve parameters, titers of all diseased and all non-diseased subjects are needed. The clinical trial dataset consists of titers measured in all diseased subjects and titers measured in a subset of non-diseased subjects. Therefore, in order to estimate PoD curve parameters from the clinical trial dataset, titers of all non-diseased subjects are generated by random sampling with replacement from immunogenicity subset 500 times[a]. For each random sample MLE is performed, resulting in 500 estimates of p'_{max}, et'_{50}, γ'. For each titer value, the median of function value of these 500 PoD curve estimates form the *median curve*. Point estimate of the PoD curve is defined as the fit of $PoD(t)$ function defined in equation (3) to *median curve* data.

To estimate CIs of PoD curve, four steps are followed:

Step 1: Titers of all non-diseased subjects are generated by random sampling with replacement from immunogenicity subset. Titers of all diseased and all non-diseased are used for estimation of PoD curve parameters. Estimates p'_{max}, et'_{50}, γ' are obtained.

Step 2: Titers of all diseased and all non-diseased subjects (generated in Step 1) are combined and bootstrapped. For each individual titer a probability of disease is calculated using the PoD curve with parameter values p'_{max}, et'_{50}, γ'. New disease status is assigned to each titer randomly using the probability of disease.

Step 3: New immunogenicity subset is selected from all new non-diseased, such that the ratio of all diseased versus non-diseased in immunogenicity subset in new data match the ratio in original data.

Step 4: Titers of all new diseased and all new non-diseased subjects are randomly sampled with replacement from new immunogenicity subset and used together with all new diseased titers for second estimation of PoD curve parameters. Estimates p''_{max}, et''_{50}, γ'' are obtained.

These steps explicitly include the population variability, between-trial variability as well as the immunogenicity subset variability and incorporate the structure of interaction between the PoD curve parameters (without assuming multivariate normal distribution).

Steps 1 to 4 are repeated 500 times[a] to yield 500 combinations of p''_{max}, et''_{50}, γ'' parameter values, defining 500 PoD curve estimates. For each titer value, the 2.5 percentile and 97.5 percentile of function values of these 500 PoD curve estimates form the 95% CI of PoD curve.

The extended method was tested using the simulation framework described in Results. As expected, the extended method for CoP-based VE estimation is as accurate as the original method and slightly less precise (depending on the size of immunogenicity subset), with good coverage probabilities of CoP-based VE CIs.

---

[a] Increasing the number of replicates did not substantially change the results (as expected for such an analysis of between-trial variability in 500 Bernoulli trials).

Supplementary Note 2: Estimation of the Confidence Interval (CI) around the PoD Curve

To estimate the CI around the PoD curve, three steps are followed:

Step 1: Titers of all diseased and all non-diseased subjects are used for estimation of PoD curve parameters as described above. Estimates p'_{max}, et'_{50}, γ' are obtained.

Step 2: Titers of all diseased and all non-diseased subjects are resampled with replacement (bootstrapped). For each individual titer a probability of disease is calculated using the PoD curve with parameter values p'_{max}, et'_{50}, γ'. New disease status is assigned to each titer randomly using the probability of disease.

Step 3: Titers of all new diseased and all new non-diseased subjects are used for second estimation of PoD curve parameters. Estimates p''_{max}, et''_{50}, γ'' are obtained.

Step 2 explicitly includes the population variability as well as between-trial variability and incorporates the structure of correlation between the PoD curve parameters (without assuming multivariate normal distribution).

Steps 1 to 3 are repeated 500 times[b] to yield 500 sets $\{p''_{max}, et''_{50}, \gamma''\}$ of parameter values, corresponding to the 500 PoD curve estimates. For each parameter, the 2.5 percentile and 97.5 percentile of the 500 estimates of that parameter yield its 95% CI. For each titer value, the 2.5 percentile and 97.5 percentile of PoD values of these 500 PoD curve estimates form the 95% CI of the PoD curve at that titer.

[b] Increasing the number of replicates did not substantially change the results (as expected for such an analysis of between-trial variability in 500 Bernoulli trials).

Supplementary Note 3: Estimation of the Confidence Interval (CI) of a CoP-Based VE

The CI of CoP-based VE is calculated using four steps. The first three steps are those described in Supplementary Note 2 for obtaining the CI for the PoD curve. Two more steps are added:

Step 4: Sample, using the standard error of the mean, the mean titer of the vaccinated group and of the control group:

$$m_{\mathrm{x}} = mean_{\mathrm{x}} + N\left(0, \frac{std_{\mathrm{x}}}{\sqrt{n_{\mathrm{x}}}}\right), \tag{10}$$

where

$mean_{\mathrm{x}}$ is the mean value of titers in group x,

std_{x} is the standard deviation of titers in group x, and

n_{x} is the number of subjects in group x.

Shifting the distribution mean, $mean_{\mathrm{x}}$, by a constant to get this mean, m_{x}, accounts for uncertainty in the population mean. Uncertainty in standard deviation is not included but could just as easily be accounted for.

Step 5: VE e'' is calculated using PoD curve with parameter values p''_{max}, et''_{50}, γ'', and using the distributions of the titer.

Steps 1 to 5 are repeated 500 times[b] to yield 500 values of CoP-based VE e''. The 2.5 percentile and 97.5 percentile of these 500 estimates form the 95% CI of CoP-based VE.

Supplementary Note 4: Simulation Results for VE and Parameter Accuracy and Precision

Supplementary Table 1. Accuracy of PoD curve parameters estimation. N = 3,000.

Simulation scenario	True VE	p_{max}					et_{50}					γ				
		mean	sd	Q1	median	Q3	mean	sd	Q1	median	Q3	mean	sd	Q1	median	Q3
A	53%	0.032	0.011	0.025	0.030	0.036	6.918	0.946	6.482	7.013	7.518	8.976	5.519	5.927	7.302	10.278
B	66%	0.032	0.013	0.024	0.030	0.036	5.985	0.873	5.532	6.071	6.549	9.173	5.64	6.000	7.349	10.495
C	69%	0.032	0.010	0.025	0.030	0.036	6.934	0.982	6.434	7.029	7.544	8.872	5.208	5.937	7.266	10.428
D	80%	0.031	0.011	0.024	0.030	0.036	6.009	0.902	5.534	6.048	6.597	9.209	5.375	6.111	7.591	10.198

Supplementary Table 2. Accuracy of PoD curve parameters estimation. N = 30,000.

Simulation scenario	True VE	p_{max}					et_{50}					γ				
		mean	sd	Q1	median	Q3	mean	sd	Q1	median	Q3	mean	sd	Q1	median	Q3
A	53%	0.030	0.002	0.029	0.030	0.032	6.934	0.232	6.772	6.929	7.091	6.851	1.029	6.000	6.560	7.523
B	66%	0.030	0.003	0.029	0.030	0.032	5.962	0.230	5.812	5.942	6.102	6.990	0.93	6.393	6.623	7.534
C	69%	0.031	0.002	0.029	0.031	0.032	6.917	0.284	6.694	6.853	7.135	6.842	0.986	6.156	6.308	7.451
D	80%	0.030	0.003	0.028	0.030	0.032	6.008	0.245	5.864	6.026	6.166	7.156	0.873	6.614	6.926	7.666

Supplementary Table 3. Accuracy of VE estimation. N = 3,000

Simulation scenario	True VE	Case-count VE, %					CoP-based VE, %				
		mean	sd	Q1	median	Q3	mean	sd	Q1	median	Q3
A	53%	51.5	15.3	42.9	53.7	62.1	53.0	7.2	48.8	53.3	57.8
B	66%	63.9	13.6	57.1	65.8	73.8	65.3	6.4	61.5	65.8	69.8
C	69%	68.0	11.3	61.6	69.3	76.1	68.6	7.1	64.3	69.2	73.9
D	80%	79.2	9.2	73.8	80.8	85.7	79.7	5.5	76.4	80.3	83.4

Supplementary Table 4. Accuracy of VE estimation. N = 30,000

Simulation scenario	True VE	Case-count VE, %					CoP-based VE, %				
		mean	sd	Q1	median	Q3	mean	sd	Q1	median	Q3
A	53%	53.3	4.4	50.6	53.4	56.2	53.6	2.2	52.0	53.6	55.1
B	66%	65.7	3.8	63.4	65.9	68.3	65.9	1.9	64.7	66.0	67.2
C	69%	69.2	3.2	67.2	69.2	71.4	69.4	2.1	67.9	69.6	71.0
D	80%	80.0	2.6	78.4	80.2	81.8	80.1	1.6	79.0	80.1	81.2

Supplementary Table 5. Estimated H3N2 antibody titers at day 50 needed to provide various levels of protection.

Probability of protection	H3N2 antibody titer level	
	estimated by Black et al.[1]	estimated by proposed method
50%	1:110	1:113
60%	1:151	1:148
70%	1:215	1:201
80%	1:330	1:300
90%	1:629	1:584

Supplementary Note 5: Accuracy of Parameter Estimation and Comparison with Precision of Case-Count Estimation

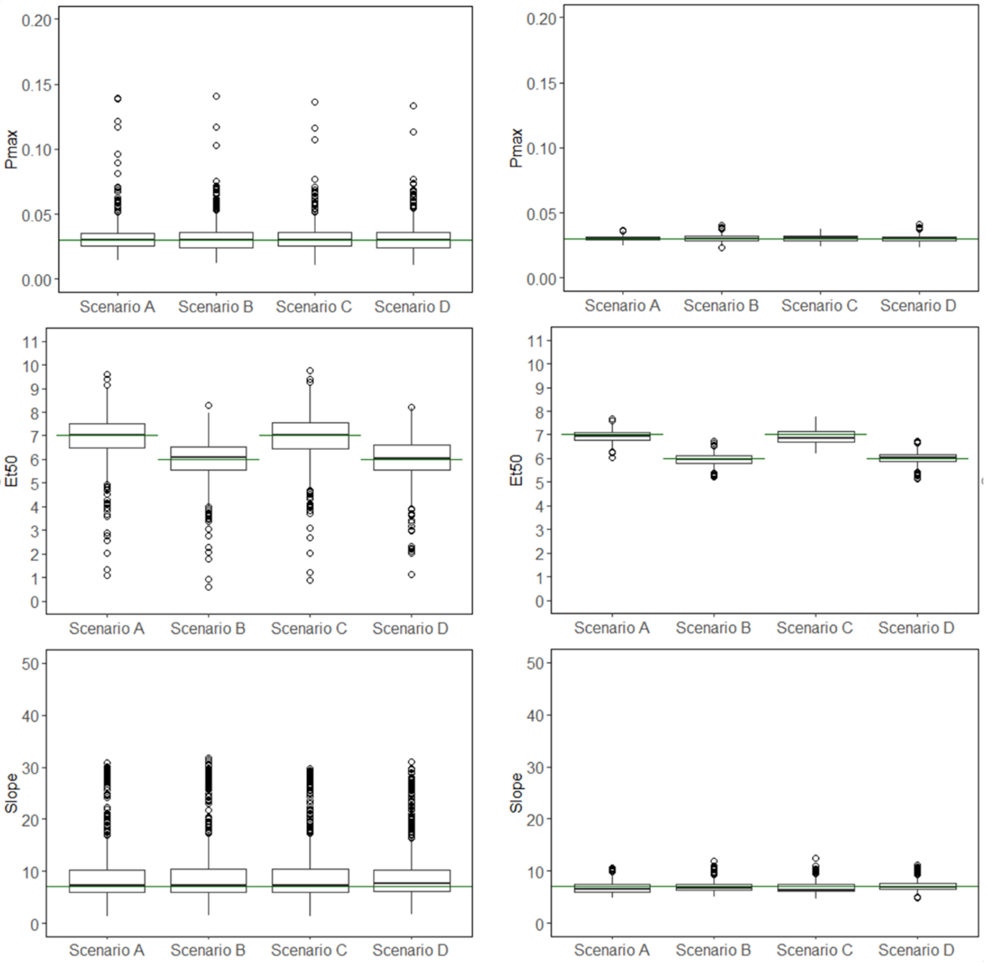

(**a**) (**b**)

Supplementary Figure 1. Accuracy of PoD curve parameters estimation. Number of subjects in simulated trial: (**a, left**) $N = 3{,}000$; (**b, right**) $N = 30{,}000$. Numerical results are provided in Supplementary Note 4, Tables 1 and 2. The outliers show that the parameter distributions are unlikely to be multivariate normal. Interpretation of box plot: center line, median; box limits, upper and lower quartiles; whiskers, 1.5x interquartile range; points, outliers. The common horizontal line represents the true VE used in the simulation.

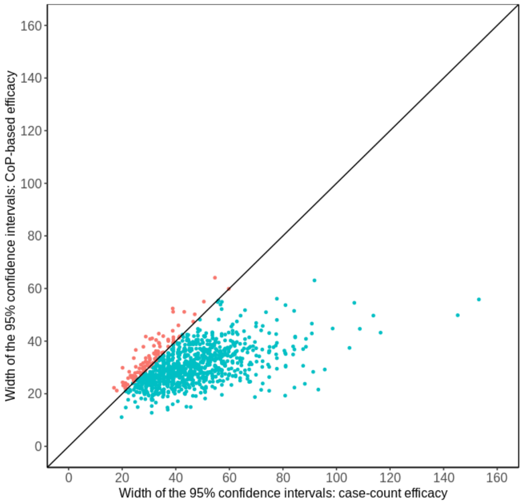

Supplementary Figure 2. Comparison of widths of CoP-based VE 95% CIs and case-count VE 95% CIs. Number of subjects in simulated trial = 3,000, simulation scenario C, true VE = 69%. Red color represents simulations in which case-count VE CI width is smaller than that of CoP-based estimate of CI.

Supplementary Note 6: Simulations Demonstrating the Advantages of PoDBAY for Informing Decisions

In order to compare PoDBAY and case-counting method of estimating VE, we simulated a number of clinical trials, calculating the false positive (FP) rates of "go" decisions and false negative (FN) rates of "no-go" decisions based on VE and its CI estimates obtained by both methods. We explored two sizes of simulated trials, corresponding by the number of virtual subjects enrolled to a typical vaccine phase 2 trial and phase 3 trial.

In the first example of a phase 2 trial, we are assuming the criterion for "go" is met if the lower bound of VE 95% CI is higher than 0% and the point estimate is above 50%. In our simulation scenarios, the true VE ranges from 0% to 100% and the vaccine is tested on 3,000 subjects (1,000 controls; 2,000 vaccinated), immunogenicity subset size is 300 subjects. The number of simulated datasets for each scenario is 1,000, with an assumed maximal probability of disease, p_{max}, of 0.03 (which corresponds, here, to a placebo incidence rate of approximately 0.02 due to the titer distribution in placebo subjects).

The second example is a phase 3 trial, assuming the criterion for "go" (to file) is met if the lower bound of VE 95% CI is higher than 25% and the point estimate is above 50%. We simulate the same vaccines as in the first example (i.e. true VE ranges from 0% to 100%). The total number of virtual subjects is 15,000 (5,000 controls; 10,000 vaccinated), and the immunogenicity subset size is 1,500 subjects. The number of simulated datasets for each scenario is 1,000, with an assumed maximal probability of disease, p_{max}, of 0.03 (and similarly to above for placebo incidence rate).

The comparison of FP and FN rates between PoDBAY and case-count are provided in Supplementary Table 6.

Supplementary Table 6. False positive rates of "go" decisions and false negative rates of "no-go" decisions based on VE and its 95% CI estimates obtained by PoDBAY and case-counting methods applied to simulated datasets. Each scenario has 1,000 simulated datasets, with 500 bootstrapped estimates (*cf.* Supplementary Note 2).

**True VE**	**Phase 2 trial (*N* = 3,000)**				**Phase 3 trial (*N* = 15,000)**			
	False positive rate (%)		False negative rate (%)		False positive rate (%)		False negative rate (%)	
	Case-count	PoDBAY	Case-count	PoDBAY	Case-count	PoDBAY	Case-count	PoDBAY
0%	0.3	0.0	-	-	0.0	0.0	-	-
10%	1.6	0.0	-	-	0.0	0.0	-	-
20%	4.3	0.0	-	-	0.0	0.0	-	-
25%	7.0	0.0	-	-	0.0	0.0	-	-
30%	10.9	0.5	-	-	0.3	0.0	-	-
40%	23.7	7.9	-	-	7.9	0.1	-	-
50%	45.8	47.6	-	-	46.8	49.8	-	-
60%	-	-	27.5	9.9	-	-	6.0	0.6
70%	-	-	10.3	1.2	-	-	0.2	0.0
80%	-	-	1.6	0.0	-	-	0.0	0.0
90%	-	-	0.0	0.0	-	-	0.0	0.0
100%	-	-	0.0	0.0	-	-	0.0	0.0

Results of phase 2 trial simulations show that when true VE is 40%, there is 3x lower chance to make unwarranted "go" decision using PoDBAY than with case-count. Similarly, when true VE is 60%, there is 3x lower chance to make unwarranted "no-go" decision when using PoDBAY. Similarly, in phase 3 the

chance of an incorrect decision is several-fold higher using case-count than using PoDBAY (when true efficacy is not close to the decision boundary). Naturally, when the true efficacy is at the decision boundary of 50%, random variability in trial results will govern any decision made, and both methods have a false positive rate approaching 50%.

The phase 3 trial simulation results also show that there is a smaller chance of making unwarranted false positive "go" decisions with PoDBAY than with case-count, with only small chance of such an error using either approach.

Based on the phase 3 trial simulation results and the assumed "go" criterion, there is only a small chance of making unwarranted decisions with both PoDBAY and case-count. In order to get FP rate of case-count 0.025 in phase 3 trial, the true VE would have to be ~36%, which corresponds to the assumed efficacy to power the phase 3 to 15,000 subjects.

Two additional examples were investigated, with simulation results demonstrating that the decisions using PoDBAY have more power while still controlling the type I ("false positive") error rate. A phase 2 trial was simulated with a decision to go to phase 3 if the lower bound of VE 95% CI is higher than 0%. The parameters of the simulated phase 2 trial are identical to those used in the phase 2 example above. A phase 3 trial was also simulated, with a decision to file ("go") if the lower bound of VE CI is higher than 25%. The parameters of the simulated phase 3 trial are identical to those used in the phase 3 example above.

The comparison of FP and FN rates between PoDBAY and case-count for these examples are provided in Supplementary Table 7. For the Phase 2 trial, the false positive rate (type I error) for a true VE=0 is expected to be 2.5 for both case-count- and PoDBAY -based decisions. Because 10,000 simulations were used, the 95% confidence interval around these values is approximately +/-0.3% (based on their being Bernoulli trials with false positive rate estimation using the proportion positive). For the phase 3 trial, the same is true when the true VE is set to 25%.

Supplementary Table 7. False positive rates of "go" decisions and false negative rates of "no-go" decisions based on VE 95% CI estimates obtained by PoDBAY and case-counting methods applied to simulated datasets. Each scenario has 1,000 simulated datasets, with 500 bootstrapped estimates (*cf.* Supplementary Note 2).

**True VE**	**Phase 2 trial (*N* = 3,000)**				**Phase 3 trial (*N* = 15,000)**			
	False positive rate (%)		False negative rate (%)		False positive rate (%)		False negative rate (%)	
	Case-count	PoDBAY	Case-count	PoDBAY	Case-count	PoDBAY	Case-count	PoDBAY
0%	2.6*	2.8*	-	-	0.0	0.0	-	-
10%	-	-	94.4	56.5	0.0	0.0	-	-
20%	-	-	86.3	7.6	0.5	0.0	-	-
25%	-	-	83.0	1.0	2.6*	2.1*	-	-
30%	-	-	75.9	0.1	-	-	92.5	59.8
40%	-	-	60.5	0.0	-	-	63.0	0.3
50%	-	-	41.2	0.0	-	-	17.3	0.0
60%	-	-	21.9	0.0	-	-	1.0	0.0
70%	-	-	8.5	0.0	-	-	0.1	0.0
80%	-	-	1.6	0.0	-	-	0.0	0.0
90%	-	-	0.0	0.0	-	-	0.0	0.0
100%	-	-	0.0	0.0	-	-	0.0	0.0

*based on 10,000 simulations where the number of bootstrapped estimates (see Supplementary Note 2) was increased to 8,000 (instead of 500)

Supplementary Note 7: Convergence of Maximum Likelihood Estimation, Limiting the Steepness of the PoD Curve, Initial Guesses of PoD Curve Parameters

The likelihood maximization algorithm can fail to converge if the number of disease cases, whose titers are used for the PoD curve estimation, is too low. Table G1 summarizes the frequency of convergence failures by number of disease cases in simulated dataset. In the 500 bootstrapped MLE estimates (see Supplementary Note 2), if at least half of the MLE estimations converge, the resulting VE is reported, and that trial simulation is considered to have a converged VE. In the tests run here (Supplementary Table 8), if even one of the 500 bootstrap MLEs succeeded, then more than half did, so the results are not sensitive to the threshold used for accepting VE convergence.

Supplementary Table 8. Convergence of VE estimation. Number of subjects in simulated trial N = 3,000, simulation scenario C, true VE = 69%, p_{max} = 0.005, 0.01; 0.02. In datasets with immunogenicity data of 15 and more diseased subjects, it is a rare event (1% or less) that the PoD curve could not be estimated.

Number of disease cases	Total number of simulations	VE convergence rate (%)
1	16	56
2	46	41
3	58	53
4	122	66
5	157	76
6	199	77
7	164	89
8	157	86
9	151	90
10	155	98
11	140	96
12	132	95
13	102	98
14	106	99
15	84	98
>16	~1000	~100

In datasets with immunogenicity data of 15 and more diseased subjects, it is a rare event (1% or less) that the PoD curve couldn't be estimated.

The setup of L-BFGS-B algorithm can also impact the convergence as well as the speed of the optimization. Dunning et al.[2] proposed to limit the steepness parameter of the probability of protection (PoP) curve. Because the β parameter of the PoP curve is analogous to the γ parameter of the PoD curve, we use the same bounds for γ parameter as described in Dunning et al.[2]

The initial guess of γ parameter is set to 6. If necessary, this can be adjusted based on visual inspection of the data.

The initial guess of p_{max} parameter is calculated as the disease rate among subjects with lowest titers (the bottom 20% of the immunogenicity dataset).

The initial guess of et_{50} is also determined using the immunogenicity dataset by (i) searching for a "middle point" between the titers of diseased and non-diseased subjects. This search is done by assuming a log normal distribution in both groups, and the titer at which the distributions (probability density functions) intersect is returned as the initial guess; if the MLE resulting from (i) does not converge, then (ii) the initial guess is taken to be the median of all of the titers (diseased and non-diseased).

Supplementary Note 8: Pronunciation Key

Preferred pronunciation of PoDBAY is "POD-bay."[3]

Supplementary Note 9: Terminology Comparison for Correlates of Protection

Reference	Term	Term used in this work and Plotkin and Gilbert, 2017[4]
WHO, 2013[5]	CoP	mechanistic or non-mechanistic CoP
WHO, 2013[5]	SoP, surrogate of protection	mechanistic CoP
Plotkin, 2008[6]	CoP	mechanistic CoP
Plotkin, 2008[6]	SoP	non-mechanistic CoP
Qin et al., 2007[7]	CoR, correlate of risk	mechanistic or non-mechanistic CoP
Qin et al., 2007[7]	different levels of SoP	mechanistic or non-mechanistic CoP: levels are de termined by association with specific statistical m ethods used for their evaluation

Supplementary Note 10: Correlates of Protection, Conditional Independence, and Goodness of Fit.

The PoDBAY framework uses a probability of disease curve independent of vaccine status (treatment group), which assumes that the vaccine's effect on the disease acts entirely through the measured immune response biomarker(s). When considered in the context of information theoretic and related approaches that use network representations of the potential influences,[8-11] the assumption (under a "causal-effects paradigm"[11]) is, formally, that the vaccine protection acts entirely through the titer. It thus assumes that the PoD relationship is independent of other factors (demographic, immunological, or experimental), so that no other factors influence the probability of a subject becoming diseased. As with any CoP analysis this cannot be proven with any experiment of finite size: CoPs and related paradigms are always used with consideration of a risk-benefit tradeoff.

Thus, although this assumption is formally (from a purely mathematical standpoint) essential to CoP-based VE estimates, a practical view of real-world applications suggests that it is incumbent upon any user of the method to have enough evidence to justify their application. This pragmatic principle has enabled the use of CoPs (often without the formal step of estimating VE) in regulatory settings including licensing (requiring the strongest evidence), as well as bridging, dose-selection, and lot-consistency (requiring somewhat less evidence).

The pragmatic approach suggested above (and described further here) is also in alignment with regulatory practice. The practice, application of "fit-for-purpose" modeling, requires objectively estimated risk-benefit tradeoff in the presence of variability.[12] The outline of this practice is that models should first be built as simply as possible, with additional factors added (often but not necessarily one-at-a-time, and potentially including interactions) if there is a scientifically valid reason to consider their potential influence. The factors are tested for their potential relevance through various statistical and information-theoretic goodness-of-fit criteria including the Akaike Information Criterion, consistency under resampling, and numerical convergence. The process can be performed in a sufficiently statistically rigorous fashion that it is the standard for determining labeling information for drugs such as, for example, if different doses are needed based on demographic factors or weight, or if a compound needs to have a more restricted indication.

In this pragmatic approach, if the dependence of an outcome on a potential influential factor is sufficiently weak that it cannot be detected, or if the factor's influence can be estimated with sufficient precision, then the resulting model can be used to provide a prediction. This effectively results from a scientific, rather than statistical criterion: an effect must be strong enough (i.e. the parameter large enough

to induce a change due to changing the covariate or predictor value) to be both measurable and scientifically/clinically relevant, so that it is relevant to the decision at hand. The criteria determining the scientific or clinical relevance are usually best established in advance of final data analyses supporting decisions (to avoid bias), and require subject matter experts, decision-makers, and other stakeholders to agree on them.

The reliability of a prediction must be considered in the context of its intended use: it depends upon the sensitivity of the test (given the available data) for that factor's influence and upon the sensitivity of the use to any resulting potential impact on results. The totality of scientific evidence needs to be considered in evaluating this reliability. For PoDBAY, that includes in vitro and in vivo (non-clinical) immunology (and other host-pathogen biology) informing the relationship between titer and protection, and, also, the completeness of clinical data with respect to potentially influential factors. The enumeration, review, or evaluation of tests for the influence of factors on models (such as establishment of conditional independence) is outside the of scope of this work, as is a systematic guidance for formally establishing a CoP.

An example of this kind of approximation is in the Results section describing the dengue vaccine analysis, in which CYD-TDV efficacy is estimated using the titers without using serostatus of subjects as a factor in the PoD curve despite the substantial dependence of the titers on serostatus. The latter dependence suggests that the PoD parameters might also be influenced by serostatus and that (with appropriate data) the estimate might be refined by its inclusion in the PoD curve – however the predictions (especially in serotype 2 where there are adequate data) are sufficiently aligned with other measurements from multiple studies that any improvement is likely to be relatively minor.

Supplementary References

1. Black, S. *et al.* Hemagglutination inhibition antibody titers as a correlate of protection for inactivated influenza vaccines in children. *Pediatr Infect Dis J* **30**, 1081-1085, doi:10.1097/INF.0b013e3182367662 (2011).

2. Dunning, A. J., Kensler, J., Coudeville, L. & Bailleux, F. Some extensions in continuous models for immunological correlates of protection. *BMC Medical Research Methodology* **15**, 1-11, doi:10.1186/s12874-015-0096-9 (2015).

3. Clarke, A. C. & Kubrick, S. YouTube video. <https://www.youtube.com/watch?v=qDrDUmuUBTo> (2020).

4. Plotkin, S. & Gilbert, P. *Correlates of Protection.* 7th ed. edn, 35-40 (Elsevier, 2017).

5. Nguipdop Djomo, P., Thomas, S. L. & Fine, P. E. M. Correlates of vaccine-induced protection: methods and implications. *World Health Organization, The Department of Immunization, Vaccines and Biologicals* (2013).

6. Plotkin, S. A. Correlates of vaccine-induced immunity. *Clinical Infectious Diseases* **3**, 401-409, doi:10.1086/589862 (2008).

7. Qin, L., Gilbert, P. B., Corey, L., McElrath, M. J. & Self, S. G. A framework for assessing immunological correlates of protection in vaccine trials. *Journal of Infectious Diseases* **9**, 1304-1312, doi:10.1086/522428 (2007).

8. Alonso, A. & Molenberghs, G. Surrogate marker evaluation from an information theory perspective. *Biometrics* **63**, 180-186, doi:10.1111/j.1541-0420.2006.00634.x (2007).

9. Alonso, A., Van der Elst, W., Molenberghs, G., Buyse, M. & Burzykowski, T. On the relationship between the causal-inference and meta-analytic paradigms for the validation of surrogate endpoints. *Biometrics* **71**, 15-24, doi:10.1111/biom.12245 (2015).

10. Fridman, A. Mixed Markov models. *Proc Natl Acad Sci U S A* **100**, 8092-8096, doi:10.1073/pnas.0731829100 (2003).

11. Joffe, M. M. & Greene, T. Related causal frameworks for surrogate outcomes. *Biometrics* **65**, 530-538, doi:10.1111/j.1541-0420.2008.01106.x (2009).

12. U.S. Department of Health and Human Services Food and Drug Administration, Center for Drug Evaluation and Research (CDER), Center for Biologics Evaluation and Research (CBER). *Population Pharmacokinetics Guidance for Industry*, <https://www.fda.gov/media/128793/download> (2019).
