# Supplementary material for: A method to estimate probability of disease and vaccine efficacy from clinical trial immunogenicity data
Source: NPJ Vaccines. 2021 Nov 4;6:133. doi: 10.1038/s41541-021-00377-6 (PMC8568947; doi:10.1038/s41541-021-00377-6)
Supplement: Supplementary file 2 — Reporting Summary [file 41541_2021_377_MOESM2_ESM.pdf]

nature research

Corresponding author(s): Jeffrey R Sachs

Last updated by author(s): Jul 19, 2021

Reporting Summary

Nature Research wishes to improve the reproducibility of the work that we publish. This form provides structure for consistency and transparency in reporting. For further information on Nature Research policies, see our Editorial Policies and the Editorial Policy Checklist.

Statistics

For all statistical analyses, confirm that the following items are present in the figure legend, table legend, main text, or Methods section.

n/a	Confirmed	
☐	☒	The exact sample size (n) for each experimental group/condition, given as a discrete number and unit of measurement
☐	☒	A statement on whether measurements were taken from distinct samples or whether the same sample was measured repeatedly
☐	☒	The statistical test(s) used AND whether they are one- or two-sided<br>*Only common tests should be described solely by name; describe more complex techniques in the Methods section.*
☐	☒	A description of all covariates tested
☐	☒	A description of any assumptions or corrections, such as tests of normality and adjustment for multiple comparisons
☐	☒	A full description of the statistical parameters including central tendency (e.g. means) or other basic estimates (e.g. regression coefficient) AND variation (e.g. standard deviation) or associated estimates of uncertainty (e.g. confidence intervals)
☐	☒	For null hypothesis testing, the test statistic (e.g. F, t, r) with confidence intervals, effect sizes, degrees of freedom and P value noted<br>*Give P values as exact values whenever suitable.*
☐	☒	For Bayesian analysis, information on the choice of priors and Markov chain Monte Carlo settings
☐	☒	For hierarchical and complex designs, identification of the appropriate level for tests and full reporting of outcomes
☐	☒	Estimates of effect sizes (e.g. Cohen's d, Pearson's r), indicating how they were calculated

Our web collection on statistics for biologists contains articles on many of the points above.

Software and code

Policy information about availability of computer code

Data collection	All software was written in and produced using R statistical software version 3.5.2. A package implementing the PoDBAY algorithm will be uploaded into the Comprehensive R Archive Network, https://cran.r-project.org/, with code, version, and persistent url location identifiers provided in compliance with the standards required by NPJ Vaccines.
Data analysis	All software was written in and produced using R statistical software version 3.5.2. A package implementing the PoDBAY algorithm will be uploaded into the Comprehensive R Archive Network, https://cran.r-project.org/, with code, version, and persistent url location identifiers provided in compliance with the standards required by NPJ Vaccines.

For manuscripts utilizing custom algorithms or software that are central to the research but not yet described in published literature, software must be made available to editors and reviewers. We strongly encourage code deposition in a community repository (e.g. GitHub). See the Nature Research guidelines for submitting code & software for further information.

Data

Policy information about availability of data

All manuscripts must include a data availability statement. This statement should provide the following information, where applicable:

- Accession codes, unique identifiers, or web links for publicly available datasets
- A list of figures that have associated raw data
- A description of any restrictions on data availability

All data that support the findings in this work are available publicly from the cited references except for the subject-level data used in the zoster vaccine analysis. The data that support the findings in that section will be made available according to the data sharing policy of Merck Sharp & Dohme Corp., a subsidiary of Merck & Co., Inc., Kenilworth, NJ, USA, which is available at http://engagezone.msd.com/ds_documentation.php. Requests for access to the clinical study data can be submitted through the Engage Zone site or via email to dataaccess@merck.com.

Field-specific reporting

Please select the one below that is the best fit for your research. If you are not sure, read the appropriate sections before making your selection.

- [x] Life sciences
- [ ] Behavioural & social sciences
- [ ] Ecological, evolutionary & environmental sciences

For a reference copy of the document with all sections, see nature.com/documents/nr-reporting-summary-flat.pdf

Life sciences study design

All studies must disclose on these points even when the disclosure is negative.

Sample size	Sample sizes ranged from 3000 to 38,543 depending on the (previously) published or simulated data set
Data exclusions	None
Replication	multiple simulations/replications
Randomization	Published data were from several randomized clinical trials. Simulations used standard pseudo-random number generators.
Blinding	The modeling analyses described in this paper used published data from several clinical trials, most of which employed blinding. The blinding status is not relevant for the current paper.

Reporting for specific materials, systems and methods

We require information from authors about some types of materials, experimental systems and methods used in many studies. Here, indicate whether each material, system or method listed is relevant to your study. If you are not sure if a list item applies to your research, read the appropriate section before selecting a response.

Materials & experimental systems

n/a	Involved in the study	
☒	☐	Antibodies
☒	☐	Eukaryotic cell lines
☒	☐	Palaeontology and archaeology
☒	☐	Animals and other organisms
☒	☐	Human research participants
☒	☐	Clinical data
☒	☐	Dual use research of concern

Methods

n/a	Involved in the study	
☒	☐	ChIP-seq
☒	☐	Flow cytometry
☒	☐	MRI-based neuroimaging
